# The FOXC2‐LAMA4 Axis Orchestrates Vasculogenic Mimicry and Immunosuppressive Niche Formation to Drive Metastatic Cascade in Renal Cell Carcinoma

**DOI:** 10.1002/advs.202516382

**Published:** 2026-01-29

**Authors:** Jiaxi Yao, Tong Xu, Chengyuan Wang, Chenyuan Wang, Junfeng Xie, Rui Zheng, Ke Wang, Xingzuo Jiang, Zewei Hu, Hongwei Jing, Lin Li, Tao Liu

**Affiliations:** ^1^ Department of Urology The First Hospital of China Medical University Shenyang P. R. China; ^2^ Department of Urology The Second Hospital of Chongqing Medical University Chongqing P. R. China; ^3^ Department of Cell Biology Key Laboratory of Cell Biology National Health Commission of the PRC and Key Laboratory of Medical Cell Biology Ministry of Education of the PRC China Medical University Shenyang P. R. China; ^4^ Department of Epidemiology School of Public Health China Medical University Shenyang Liaoning P. R. China; ^5^ Key Laboratory of Major Chronic Diseases of Nervous System of Liaoning Province Health Sciences Institute of China Medical University Shenyang P. R. China; ^6^ Department of Rehabilitation Shengjing Hospital of China Medical University Shenyang P. R. China

**Keywords:** clear cell renal cell carcinoma, FOXC2, metastatic microenvironment, single‐cell multi‐omics, vasculogenic mimicry

## Abstract

The aggressive metastatic propensity of advanced clear cell renal cell carcinoma (ccRCC) originates from intratumoral heterogeneity. Through integrated single‐cell and spatial multi‐omics profiling, we identified a FOXC2^+^ tumor subpopulation endowed with vasculogenic mimicry capability as pivotal effector cells driving metastasis. Mechanistically, the transcription factor FOXC2 binds the promoter region of LAMA4 to activate its expression, initiating metastatic cascades via vasculogenic mimicry remodeling. In orthotopic lung metastasis models, FOXC2^+^ tumor cells leveraged LAMA4 to reshape the pulmonary metastatic niche, thereby reinforcing distant metastatic dissemination. Tumor‐secreted LAMA4 engaged macrophage surface receptor ITGA6 to trigger GATA3 activation and reprogram macrophages toward a pro‐metastatic and immunosuppressive phenotype. Disruption of LAMA4‐ITGA6 binding substantially attenuated FOXC2‐LAMA4‐mediated metastatic burden. These results reveal a novel mechanism by which FOXC2^+^ tumor cells promote metastasis in advanced ccRCC and further establish the therapeutic potential of targeting FOXC2‐LAMA4 in blocking the metastatic cascade of ccRCC.

## Graphical abstract

Advanced clear cell renal cell carcinoma metastasis is driven by a FOXC2^+^ tumor subpopulation. These cells execute vasculogenic mimicry for dissemination and secrete LAMA4. LAMA4 engages macrophage ITGA6, activating the STAT6/GATA3 axis to polarize macrophages into a pro‐metastatic, immunosuppressive phenotype. Disrupting the LAMA4‐ITGA6 interaction reduces metastatic burden, identifying this axis as a promising therapeutic target for inhibiting ccRCC metastasis.

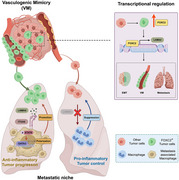

## 1 Introduction

Clear cell renal cell carcinoma (ccRCC), the most aggressive malignancy of the urinary system, accounts for 75%–80% of all renal cancer cases [1]. Epidemiological data indicate that approximately 30% of newly diagnosed patients already present with distant metastasis, leading to a significant decline in survival rates [2]. Although anti‐angiogenic therapies (AAT) such as sunitinib can prolong overall survival, most patients inevitably develop drug resistance and metastasis [3]. Recent single‐cell sequencing studies have revealed marked heterogeneity in ccRCC, with its robust phenotypic plasticity closely associated with tumor metastasis [4]. Thus, investigating the tumor microenvironment (TME) of ccRCC to uncover the mechanisms underlying therapeutic failure and metastasis remains an urgent scientific challenge [5].

Tumor metastasis is a spatially dynamic cascade process involving multiple steps, including detachment from the primary site and colonization of target organs [6, 7]. The high heterogeneity of primary tumors enables certain cell subpopulations to undergo epithelial‐mesenchymal transition (EMT), enhancing their plasticity and conferring metastatic potential [5, 6]. Studies have confirmed that EMT is a critical prerequisite for tumor cells to acquire vasculogenic mimicry (VM) capability [8, 9]. During this process, these cells can mimic endothelial cell behavior and self‐organize to form microvascular structures independent of the original vascular endothelium [9]. This unique blood supply system not only evades the endothelial targets of conventional AATs, leading to drug resistance, but also directly connects tumor cells to the circulatory system, facilitating their detachment as disseminated tumor cells and promoting metastasis [9, 10]. Notably, while VM has been observed in various cancers and is implicated in metastasis and drug resistance [10, 11], the intrinsic molecular regulatory network governing VM formation in ccRCC and its co‐evolutionary mechanisms with the TME remain unclear.

Remodeling of the metastatic niche microenvironment represents another critical barrier to tumor colonization [12]. Recent studies demonstrate that TREM2^+^ macrophages in the metastatic niche significantly suppress the function of cytotoxic immune cells, establishing an “immune‐exempt niche” through multiple mechanisms to promote the growth and activation of metastatic tumor cells [13, 14]. Intriguingly, the polarization of these macrophages may be driven by secretory factors from metastatic tumor cells, though the precise regulatory mechanisms remain elusive [15].

In this study, we integrated single‑cell RNA sequencing (scRNA‐seq), spatial transcriptomics (ST), and multi‑omics analyses to systematically decipher the VM‑mediated metastasis cascade in ccRCC. We defined a distinct subpopulation of FOXC2‑positive tumor cells that plays a pivotal role in ccRCC progression. Mechanistically, FOXC2 acts as a key transcriptional regulator that directly upregulates LAMA4, thereby driving VM formation. Furthermore, secreted LAMA4 engages ITGA6 on target cells, triggering STAT6 phosphorylation and subsequent GATA3 activation, which in turn polarizes TREM2^+^CD206^+^ macrophages within the metastatic niche. This signaling cascade remodels the immunosuppressive microenvironment and substantially promotes metastatic outgrowth. By addressing the clinical challenges of advanced metastasis and AAT resistance in ccRCC, our work elucidates the spatiotemporal dynamics of the FOXC2–LAMA4 axis in promoting VM‑mediated metastatic cascades, providing novel mechanistic and therapeutic insights from the perspective of tumor cell‑derived VM.

## 2 Materials and Methods

### 2.1 Patients and Ethics Statement

For scRNA‐seq data of 12 tumor tissues and 2 adjacent noncancerous tissues of ccRCC patients, we downloaded from public Gene Expression Omnibus (GEO) datasets (GSE159115, GSE156632, GSE152938) for subsequent analysis [16, 17, 18]. ST data of ccRCC were downloaded from the GSE175540 GEO dataset [19].

Samples include matched pairs of fresh ice‐frozen ccRCC and adjacent non‐cancerous tissue samples (n = 18); formalin‐fixed paraffin‐embedded samples from primary ccRCC without metastasis tissue samples (n = 16) and distant metastasis tissue samples (n = 15). Samples were obtained from the Department of urology of the First Affiliated Hospital of China Medical University.

This study was conducted in strict accordance with the ethical principles outlined in the Declaration of Helsinki (World Medical Association) and was approved by the Medical Ethics and Human Clinical Trial Committee of China Medical University (Approval No. 2024‐235). Written informed consent was obtained from all patients or their legal guardians for the use of their tissue samples. All animal experiments adhered to the ARRIVE guidelines and were performed following protocols approved by the Institutional Animal Care and Use Committee (IACUC) of China Medical University (Approval No. CMU20241848).

### 2.2 RNA‐seq Assay

Total RNA was extracted from samples using RNAiso Plus reagent (Cat# 9108, Takara, Japan). RNA integrity and quality were assessed using an Agilent 2100 Bioanalyzer (Agilent Technologies, Palo Alto, CA, USA). Eukaryotic mRNA was enriched from total RNA through poly(A) selection with Oligo(dT) magnetic beads. The purified mRNA was fragmented into short segments using fragmentation buffer and subsequently reverse‐transcribed into cDNA using the NEBNext Ultra RNA Library Prep Kit for Illumina (NEB #7530, New England Biolabs, Ipswich, MA, USA). The cDNA fragments were subjected to end repair, 3′‐end adenylation, and ligation to Illumina sequencing adapters. The ligated products were purified using AMPure XP Beads (1.0X, Beckman Coulter, USA) and amplified by polymerase chain reaction (PCR). The final cDNA library was quantified and sequenced on an Illumina NovaSeq 6000 platform (Illumina, San Diego, CA, USA) by GeneDenovo Biotechnology Co. (Guangzhou, China).

### 2.3 CUT&Tag Assay

The CUT&Tag experiment was performed using the Hyperactive Universal CUT&Tag Assay Kit (Vazyme, Cat# TD904, China) following the manufacturer's protocol with minor modifications. Briefly, cells were collected at room temperature and incubated with activated ConA Beads Pro for 10 min at room temperature. After brief centrifugation, the supernatant was discarded, and the cell‐bead complexes were resuspended in Antibody Buffer containing a primary antibody against FOXC2 (Abcam, Cat# ab5060, UK; 1:100 dilution) and incubated overnight at 4°C. The beads were then magnetically captured, washed, and incubated with a secondary antibody (ABclonal, Cat# AS031, China; 1:100 dilution) in Dig‐wash Buffer for 1 h at room temperature. Following another wash, the complexes were incubated with pA/G‐Tn5 transposase (provided in the kit) for 1 h at room temperature. After washing with Dig‐300 Buffer, DNA was fragmented by adding a 10% SDS solution containing DNA Spike‐in, and the supernatant containing released DNA was collected via magnetic separation. DNA was purified using DNA Extract Beads Pro (Vazyme, China) and amplified by PCR with indexed adapter primers (Vazyme, Cat# TD204). The final library was size‐selected and purified using VAHTS DNA Clean Beads (Vazyme, Cat# N411) and quantified for sequencing.

### 2.4 scRNA‐seq Data Preprocessing, Integration, and Identification

We used Seurat version 4.0.2 for scRNA‐seq data analysis [20]. The data quality control adopted the following standards: (1) the number of expressed genes was less than 300 or more than 4000; (2) the proportion of mitochondrial gene expression was greater than 15%. If one of the above conditions was met, the cells were considered to be low‐quality cells and were excluded.

We separately performed data normalization, identification of highly variable genes, and data scaling on 14 datasets through SCTransform. Subsequently, we controlled for batch effects using the CCA method. We applied SelectIntegrationFeatures to select 3000 anchor features for downstream integration, ran PrepSCTIntegration and FindIntegrationAnchors to identify anchor genes, and finally performed data integration. We used the RunPCA function to reduce the dimensionality of the scRNA‐Seq dataset using the first 50 principal components. Primary cell clusters were identified by the FindClusters function. Data were visualized using uniform manifold approximation and projection (UMAP) plots.

To identify cell types, we analysed differentially expressed genes (DEGs) within each cluster using FindAllMarkers. DEGs from each cluster were compared to cell‐type marker genes previously described in the literature to assign cell identity.

### 2.5 Estimation of Copy Number Variation (CNVs) in Cancer Cells

To identify large‐scale chromosomal copy number alterations, we used the InferCNV package to calculate the CNV levels of cells. We randomly sampled 800 T cells and 800 myeloid cells as a reference group (considered diploid cells) and performed CNV inference on all epithelial cells. Subsequently, we extracted the CNV matrix of reference cells and epithelial cells for k‐means clustering, and nonmalignant epithelial cells and reference cells were clustered into the same cluster and had the lowest CNV levels. After removing nonmalignant epithelial cells, 13151 cancer cells were finally obtained and reclustered into 7 clusters for analysis of cellular heterogeneity.

### 2.6 Single Cell Weighted Gene Co‐Expression Network Analysis (scWGCNA)

The scWGCNA workflow was used to perform WGCNA for single‐cell datasets [21]. The performance of vanilla WGCNA on single‐cell data is limited due to the inherent sparsity of scRNA‐seq data [22]. To account for this, scWGCNA has a function to aggregate transcriptionally similar cells into pseudobulk metacells before running the WGCNA pipeline. The Construct_metacells function takes a highly variable gene to construct the pseudobulk metacell (k = 100) and generates a new Seurat object for subsequent WGCNA. After constructing the adjacency matrix, we chose the soft‐thresholding power (β = 12) to obtain the topological overlap matrix (TOM). Genes were grouped using average linkage hierarchical clustering based on the high similarity of coexpression relationships.

### 2.7 Gene Regulatory Network Analysis

pySCENIC is a new computational method to map gene regulatory networks and identify stable cell states by evaluating the activity of each cell from scRNA‐seq data [23]. To measure the different regulon activities among cell clusters, pySCENIC was performed on all cancer cells. We calculated the average score for each cell type and selected the top 5 differentially activated transcription factors in each group to generate heatmaps. Only regulons significantly upregulated were involved in subsequent analysis.

### 2.8 Pathway Enrichment Analysis

DEGs were calculated through the function FindMarkers in the Seurat package. ClusterProfiler (version 4.2.1) was applied to perform GO and KEGG enrichment of the genes in scWGCNA modules [24]. GSVA was conducted with the GSVA package (1.42.0) using the gene sets from MSigDB.

### 2.9 ST Data Analysis

We used the Seurat package to analyse the matrices of gene dots generated by ST data processing. Signature scoring based on scRNA‐seq and MSigDB data was performed with the AddModuleScore function. Spatial feature expression plots were generated with the SpatialFeaturePlot function. We applied RCTD to deconvolve spatial transcriptomic data, which allows for the quantification of spatial and local environmental effects on cell type‐specific gene expression.

### 2.10 Correlation to Bulk RNA‐seq

TCGA KIRC mRNA expression (FPKM) data and clinical information were downloaded from the Xena browser. To construct the CIBERSORTx signature matrix of cancer cells, we ran the Create Signature Matrix module with the scRNA expression matrix from our dataset as a reference matrix [25]. Using the resulting signature matrix, deconvolution was performed on the TCGA‐KIRC cohorts in relative mode with S‐mode batch correction and quantile normalization disabled. Kaplan–Meier analysis was performed to evaluate the prognostic value of different cell clusters in ccRCC progression.

### 2.11 Cell Culture

Human clear cell renal cell carcinoma (ccRCC) 786‐O and OSRC‐2, human monocytic THP‐1, and human umbilical vein endothelial cells (HUVECs) were obtained from the American Type Culture Collection (ATCC, Manassas, VA, USA). The 786‐O and THP‐1 cells were cultured in RPMI 1640 medium (Gibco, C11875500BT) with 10% fetal bovine serum (Biological Industries, 04‐001‐1ACS). OSRC‐2 cells were cultured in high‐glucose DMEM (Gibco, C11995500BT) with 10% fetal bovine serum (Biological Industries, 04‐001‐1ACS). HUVECs were cultured in EBM‐2 (Lonza, CC‐3156) supplemented with 10% fetal bovine serum (Biological Industries, 04‐001‐1ACS). All cell lines were cultured at 37°C in a humidified 5% CO2 atmosphere (Thermo Fisher Scientific).

### 2.12 Lentiviral Transduction

Lentiviral constructs encoding human FOXC2 short hairpin RNAs (shRNAs), mouse Flag‐Vector, Flag‐Foxc2, nonsilencing control shRNA (shCtrl), and shFoxc2 were commercially generated (Shanghai Genechem Co., Ltd.). Cells were transduced with lentiviral particles for 24 h, followed by selection with puromycin (1 µg/mL) for 24–48 h.

### 2.13 Quantitative RT–PCR (qRT–PCR)

Total RNA was extracted with RNAiso Plus reagent (Takara, 9108) according to the manufacturer's instructions. For cDNA synthesis, total RNA was reverse transcribed to cDNA using the PrimeScript RT kit (TAKARA, RR047A). The resulting cDNA was diluted 10 times before use. Quantitative real‐time PCR (qRT–PCR) was performed on an Applied Biosystems QuantStudio 6 Flex Cycler (ThermoFisher Scientific) using SYBR Mix (TAKARA). The PCR primers are listed in (Table S1).

### 2.14 Western Blot Analysis

Cells were lysed in NP‐40 buffer (20 mm Tris‐HCl [pH 7.5], 50 mm NaCl, 10% glycerol, 1% NP‐40) containing protease inhibitors (Dingguo Biology). Protein concentrations were determined by BCA assay (Beyotime, P0010S). Proteins were separated by SDS‐PAGE and transferred to PVDF membranes (Bio‐Rad, 1620177). After blocking with 5% BSA (Beyotime, ST023), membranes were incubated overnight at 4°C with primary antibodies, followed by appropriate HRP‐conjugated secondary antibodies. Protein bands were visualized using ECL substrate (Pierce) and quantified using a Lumat LB 9507 imaging system (Berthold Technologies).

Primary antibodies included: anti‐FOXC2 (Abcam, ab5060; 1:1000), anti‐E‐cadherin (CST, 14472S; 1:1000), anti‐N‐cadherin (CST, 13116S; 1:1000), anti‐vimentin (CST, 5741T; 1:1000), anti‐TGFβ (Proteintech, 21898‐1‐AP; 1:1000), anti‐CD206 (Cohesion, CPA1765; 1:1000), anti‐TREM2 (ABmart, M079355), anti‐LAMA4 (Abmart, PK07715; 1:1000), anti‐GATA3 (CST, 5852; 1:1000), anti‐STAT6 (Proteintech, 82630‐1‐RR; 1:1000), anti‐pSTAT6 (CST, 56554; 1:1000), anti‐GAPDH (Proteintech, 60004‐1‐Ig; 1:10000).

### 2.15 Co‐Immunoprecipitation (Co‐IP)

Co‐IP was performed using an IP lysis buffer supplemented with protease and phosphatase inhibitors. Total protein lysate (2 mg) was incubated overnight with anti‐LAMA4 (Santa Cruz Biotechnology, sc‐130541) and Protein A/G agarose beads. Precipitated proteins were analyzed by Western blotting.

### 2.16 Wound Healing Assay

Cells were dissociated into single cells and plated on 6‐well plates. When the cells attached, the cell cultures were scratched using a pipette tip. The cells were photographed at 6 and 12 h after wounding. Data were analysed by ImageJ. The wound healing rate was calculated as follows: (original migration area—scratch migration area after healing)/(original migration area) x 100%.

### 2.17 Cell Migration Assay

A total of 2 × 10^4^ cells were transferred into the upper chamber of 24‐well Transwell inserts (Corning, CLS3464) with serum‐free medium, and 500 µL complete medium (10% FBS) was added to the lower chamber. When a certain number of cells were observed in the lower chamber, the upper chamber was removed. The cells on the bottom side were fixed with ethyl alcohol for 20 min and stained with crystal violet for 30 min. Stained cells were photographed by microscope, and the number of cells was analysed by ImageJ.

### 2.18 Vasculogenic Tube Formation Assay

Fifty microlitres of Matrigel (Corning, 356234) was placed into 96‐well plates and kept at 37°C for 30 min. 786‐O cells (1.5^*^10^4^/mL) were seeded into the wells. Tube formation was detected under a microscope (Olympus AX‐70) after 6 h.

### 2.19 Immunohistochemistry (IHC) and Periodic Acid Schiff (PAS) Staining

Tissues were embedded in paraffin and cut into 4‐µm‐thick slices. The sections were dewaxed and rehydrated with xylene and ethanol. Antigen retrieval was performed by boiling using citrate antigen retrieval solution. The sections were blocked with serum for 1 h and then incubated overnight with primary antibodies against FOXC2 (1:200; Bioss, bs‐8730R) and CD31 (1:200; Cohesion Biosciences, CPA9724) at 4°C. After incubation with a secondary antibody (MXB, KIT‐9902), DAB (MXB, DAB‐2031) was used for colour development and counterstained with haematoxylin. Sections were observed and photographed using a microscope. The PAS staining was performed following the manufacturer's protocol (Solarbio, G1281). The slides were incubated for 8 min at room temperature with PAS, and incubated for 20 min at room temperature with Schiff's Reagent after washing.

### 2.20 Chromatin Immunoprecipitation (ChIP)

We analysed transcription factor binding sites in the LAMA4 promoter using the JASPAR website. ChIP assays were performed as the manufacturer's protocol (Abcam, ab500). Cells were cross‐linked in 1.1% formaldehyde for 10 min and quenched by glycine. Sonicated DNA was acquired by lysis and sonication. Chromatin and antibodies were incubated overnight with rotation at 4°C. Beads and DNA‐protein complexes were mixed and rotated for 1 h at 4°C, then washed with 1x ChIP buffer. DNA was purified by the DNA purifying slurry and then used for quantitative PCR. The PCR primers are listed in (Table S1).

### 2.21 Dual‐Luciferase Assays

Cells were co‐transfected with the FOXC2 plasmid and LAMA4 promoter reporter constructs (pGL3 vector). Luciferase assay was conducted using a luciferase assay kit (Promega, E1910) 24 h after transfection. Luciferase readings were normalized to total protein content. Each parameter was studied in triplicate, and the experiment was repeated at least three times.

### 2.22 Transient Transfection

When cells rich 50% density, the plasmid was mixed with HiGene (APPLYGEN, C1506‐1) based on the manufacturer's guide and incubated for 15 min at room temperature, and then added into cell culture dish. Cells were harvested at 36–48 h after transfection.

### 2.23 Animal Experiments

BALB/c mice (4–5 weeks old, 18–20 g) were purchased from BEIJING HFK BIOSCIENCE CO (Beijing, China). 5^*^10^5^ RENCA cells with stable expression of Flag Vector, Flag‐Vector+shLama4, Flag‐Foxc2+shCtrl, Flag‐Foxc2+shLama4 were injected into the left renal capsule of BALB/c mice. At the experimental endpoint (4 weeks post‐injection), mice were euthanized, and tumors were excised for measurement.

Renca‑luc Foxc2^OE^ cells were orthotopically implanted into the kidneys of BALB/c mice. Treatment began on day 7 post‑implantation. Sunitinib (MedChemExpress, Cat# HY‑10255A) was formulated in 5% DMSO plus 95% corn oil and administered daily by oral gavage at a dose of 40 mg/kg. Treatment cycles consisted of 5 days of dosing followed by 2 days off, repeated for a total of five cycles. Anti‑ITGA6 antibody (Biolegend, Cat# 313602, Clone GoH3) was injected intravenously at 10 mg/kg once weekly. Four experimental groups (n = 10 per group) were included: Control (solvent + Rat‑IgG), Sunitinib monotherapy (Sunitinib + Rat‑IgG), anti‑ITGA6 monotherapy (solvent + anti‑ITGA6 Ab), and combination therapy (Sunitinib + anti‑ITGA6 Ab).

To enable extended survival observation, a low‑dose experimental lung metastasis model was established [26]. Renca‑luc Foxc2^OE^ cells (0.5 × 10^5^ cells in 100 µL PBS) were injected via the tail vein. Treatment started on day 7 post‑injection. Mice were randomly assigned to two groups (n = 5 per group): a control group receiving Sunitinib (same regimen as above) plus Rat‑IgG, and a combination group receiving Sunitinib plus weekly anti‑ITGA6 Ab. Lung metastases were monitored by in vivo bioluminescence imaging. At the endpoint, lung tissues were collected for H&E staining.

In accordance with ethical guidelines, mice were euthanized when tumor volume exceeded 1000 mm^3^, and survival was monitored accordingly. Mice received intraperitoneal injections of luciferin substrate (150 mg/kg) and were imaged weekly using a Carestream MS FX Pro in vivo imaging system (Carestream Health, Rochester, NY, USA). Bioluminescence signals were quantified using LivingImage software (PerkinElmer, Waltham, MA, USA). Tumor volume was calculated as: volume = length ^*^ width ^*^ width/2. Survival was recorded throughout the study for Kaplan‑‐Meier analysis.

### 2.24 Single‐Cell RNA Sequencing Workflow of Animal Models

Lung tissues from the FOXC2^OE^ LAMA4^NC^ and FOXC2^OE^ LAMA4^KD^ groups were harvested at the experimental endpoint for single‐cell sequencing. Fresh lung tissues were mechanically dissociated into single‐cell suspensions using enzymatic digestion. Cell suspensions were filtered through 70‐µm strainers, washed twice with PBS, and centrifuged at 300 × g for 5 min. CD45^+^ cells were positively selected using the EasySep Mouse CD45 Positive Selection Kit (StemCell Technologies, Catalog #18945) according to the manufacturer's protocol. To ensure balanced representation of immune and non‐immune compartments, CD45^+^ and CD45^−^ cells were combined at a 3:1 ratio prior to sequencing. For each experimental group, lung tissues from three independent biological replicates were pooled to minimize inter‐individual variability. Single‐cell suspensions were loaded onto a Chromium Controller (10x Genomics) targeting 10 000 cells per sample. Libraries were constructed using the Chromium Single Cell 3′ Reagent Kit v3.1 (10x Genomics) and sequenced on an Illumina NovaSeq 6000 platform. The Cell Ranger software was obtained from the 10x Genomics website. Alignment, filtering, barcode counting, and UMI counting were performed with the cellranger count module to generate a feature‐barcode matrix and determine clusters.

### 2.25 Preparation and Culture of Bone Marrow‐Derived Macrophages from Mice

Bone marrow cells were isolated from 6‐week‐old C57BL/6 mice and cultured in RPMI 1640 medium supplemented with 5% fetal bovine serum (FBS), 20 ng/mL M‐CSF Recombinant Protein (PeproTech, 315‐02), and 100 U/mL penicillin‐streptomycin [27]. On day 4, half of the medium was replaced with fresh culture medium. After 7 days, the adherent cells were harvested as immature bone marrow‐derived macrophages (BMDMs). In parallel, immature BMDMs were divided into three groups and treated for 24 h: an untreated control, a positive control stimulated with 25 ng/mL IL‐4 (SinoBiological, 51084‐MNAE), and an experimental group treated with 25 ng/mL LAMA4 (Origene, TP724250). Cells were then harvested, washed, and prepared as single‐cell suspensions.

### 2.26 Flow Cytometric Analysis

THP‐1 monocytes were differentiated into M0 macrophages by treatment with 100 ng/mL phorbol 12‐myristate 13‐acetate (PMA; Selleck, S7791) for 48 h. The differentiated macrophages were then stimulated for 24 h with 25 ng/mL recombinant human LAMA4 (Origene, TP761994). Untreated cells and those treated with 25 ng/mL IL‐4 (PeproTech, 200‐04) served as the negative and positive controls, respectively. After stimulation, cells were collected, washed with PBS, and detached using non‐enzymatic cell dissociation buffer, followed by incubation for 30 min at 4°C with anti‐CD68 (clone Y1/82A, BioLegend) and anti‐CD206 (clone 19.2, eBioscience).

BMDMs staining was performed in the dark at 4°C for 30 min using fluorochrome‐conjugated antibodies against CD11b (clone M1/70, eBioscience), F4/80 (clone BM8, eBioscience), and CD206 (clone MR6F3, eBioscience). After one wash with buffer, cells were resuspended in an appropriate volume for flow cytometry.

To examine the role of LAMA4 in the metastatic tumor microenvironment, we prepared single‐cell suspensions from lung metastases of FOXC2^OE^ LAMA4^NC^ and FOXC2^OE^ LAMA4^KD^ mice. Following euthanasia, lung tissues were collected, minced, and digested in HBSS buffer containing 1 mg/mL collagenase IV (Gibco, 17104019) and 20 µg/mL DNase I (Sigma, 10104159001) for 2 h at 37°C with shaking. The digested tissue was filtered through a 100 µm strainer and centrifuged at 2000 rpm for 5 min at 4°C. Red blood cells were lysed with ACK buffer, and viable cells were counted. Single‐cell suspensions were stained in the dark at 4°C for 30 min using the following antibodies diluted per manufacturer's instructions: L/D eFluor506 (Invitrogen, 65‐0866‐14), anti‐CD45 FITC (clone 30‐F11, eBioscience), anti‐F4/80 eFluor450 (clone BM8, Invitrogen), anti‐CD11c PerCP‐Cy5.5 (clone N418, eBioscience), anti‐CD11b APC (clone M1/70, BioLegend), anti‐Trem2 PE (clone 6E9, BioLegend), anti‐CD8a APC‐eFluor780 (clone 53‐6.7, Invitrogen), and anti‐PD‐1 PE (clone J43, Invitrogen).

All samples were acquired on a BD FACS Calibur flow cytometer (BD Biosciences), and data were analyzed using FlowJo software (version 10.0.5) in accordance with established guidelines.

### 2.27 Statistical Analysis

Statistical analyses were performed using R (v4.1.3) and GraphPad Prism (v10.0). Data are presented as mean ± SD unless otherwise specified. For comparisons, two‐tailed Student's *t*‐tests (two groups) or ANOVA were used, with *P*<0.05 considered significant. Survival differences were assessed by the Kaplan–Meier/Log‐rank test. For high‐throughput data (e.g., scRNA‐seq), multiple testing correction was applied using the Benjamini–Hochberg FDR method (*q*<0.05). Specific sample sizes (n) for each experiment are provided in the corresponding figure legends or methods.

## 3 Results

### 3.1 scRNA‐seq Reveals the Tumor Microenvironment Heterogeneity in Renal Cell Carcinoma

**FIGURE 1:**
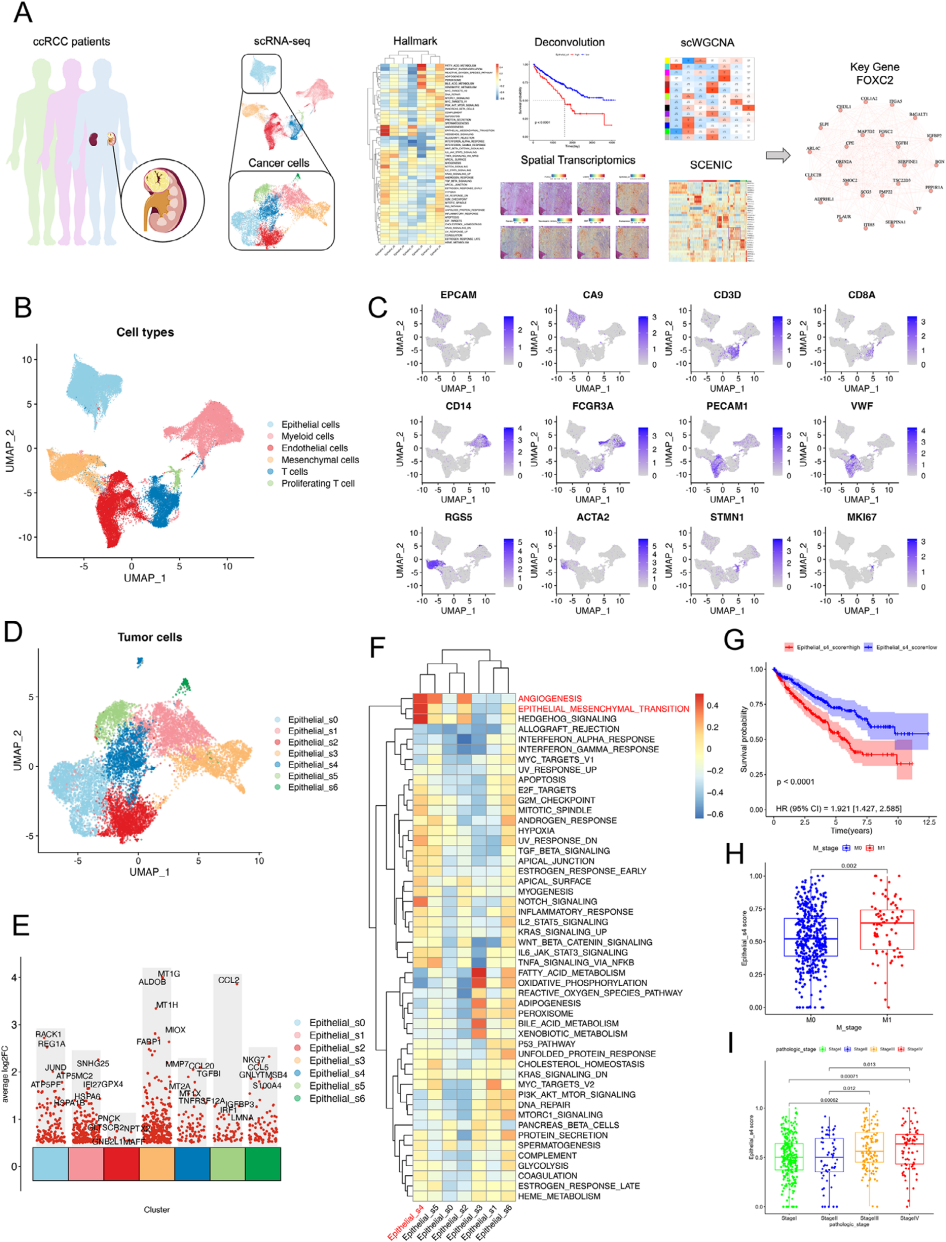
Identification of the Epithelial_s4 cell subpopulation closely associated with angiogenesis and metastasis in ccRCC via scRNA‐seq technology. (A) Bioinformatics analysis workflow. (B) Dimensionality reduction, clustering, and identification of all single cells from 12 renal carcinoma tissue samples. (C) FeaturePlot showing marker gene expression across cell subpopulations. (D) Dimensionality reduction and clustering of malignant epithelial cells into 7 subpopulations. (E)Top 5 highly expressed genes in each subpopulation. (F) GSVA analysis reveals significant enrichment of angiogenesis and epithelial‐mesenchymal transition (EMT) pathways in the Epithelial_s4 subpopulation. (G) Deconvolution of tumor cell subpopulations in the TCGA‐KIRC cohort using CIBERSORTx. Patients with a higher proportion of the Epithelial_s4 subpopulation exhibit significantly reduced overall survival rates. (H) Epithelial_s4 signature scores are higher in metastatic renal carcinoma. (I)Differences in Epithelial_s4 subpopulation signature scores across renal carcinoma stages.

This study first dissected the heterogeneity of the TME in ccRCC at single‐cell resolution. Using 10x Genomics scRNA‐seq on fresh tumor tissues from 12 ccRCC patients [16, 18, 19], we obtained 59 566 high‐quality single cells (Figure 1A). After quality control and batch‐effect correction (Figure S1A,B), the cells were clustered into six major subsets (Figure 1B) and annotated based on canonical marker genes: epithelial cells (EPCAM, CA9), T cells (CD3D, CD8A), myeloid cells (FCGR3A, CD14), endothelial cells (PECAM1, VWF), stromal cells (RGS5, ACTA2), and proliferating cells (STMN1, MKI67) (Figure 1C; Figure S1C).

CNV analysis of epithelial cells revealed substantial inter‐ and intra‐tumoral heterogeneity in ccRCC (Figure S2A–C). Notably, nearly all tumor cells exhibited chromosome 3p deletion (encompassing key driver genes such as VHL, PBRM1, SETD2, and BAP1), with subsets showing concurrent chromosome 5 amplification or chromosomes 13/14 deletions, suggesting polygenic cooperation in tumor progression [28]. To distinguish malignant from non‐malignant epithelial cells, we performed k‐means clustering of CNV profiles (Figure S2B). The results demonstrated that normal cells (T cells, myeloid cells) and a subset of low‐CNV epithelial cells (kmeans_class 4) were grouped together (Figure S2C). Finally, by excluding kmeans_class 4 epithelial cells, we obtained high‐confidence tumor cells for downstream analysis.

### 3.2 Functional Heterogeneity of Tumor Cell Subpopulations Reveals Key Subtypes Driving ccRCC Malignant Progression

To further dissect the transcriptional heterogeneity of malignant cells in ccRCC, we performed subclustering analysis on the identified tumor cells and delineated seven distinct subpopulations (Figure 1D,E). These subpopulations exhibited significant inter‐patient variability in their proportions (Figure S2D), suggesting individualized differences during tumor progression. Gene Set Variation Analysis (GSVA) based on the HALLMARK gene sets revealed marked functional divergence among these subpopulations (Figure 1F). Notably, the Epithelial_s4 subpopulation demonstrated coordinated activation of angiogenesis and EMT pathways, both of which are strongly associated with poor prognosis in ccRCC.

To validate the clinical relevance of the Epithelial_s4 subpopulation, we employed the CIBERSORTx algorithm to deconvolute bulk transcriptomic data from the TCGA‐KIRC cohort and quantify its abundance in tumor tissues. Survival analysis demonstrated that patients with a high proportion of Epithelial_s4 cells had significantly shorter overall survival (Figure 1G; Figure S2E–J). Furthermore, an increased Epithelial_s4 fraction was significantly correlated with higher risks of distant metastasis (Figure 1H) and advanced pathological stages (Figure 1I). These findings collectively suggest that Epithelial_s4 represents a key driver subpopulation in ccRCC malignant progression, highlighting its critical clinical significance.

### 3.3 Spatiotemporal Transcriptomics Reveals the Pivotal Role of the Epithelial_s4 Subpopulation in Driving VM in ccRCC

**FIGURE 2:**
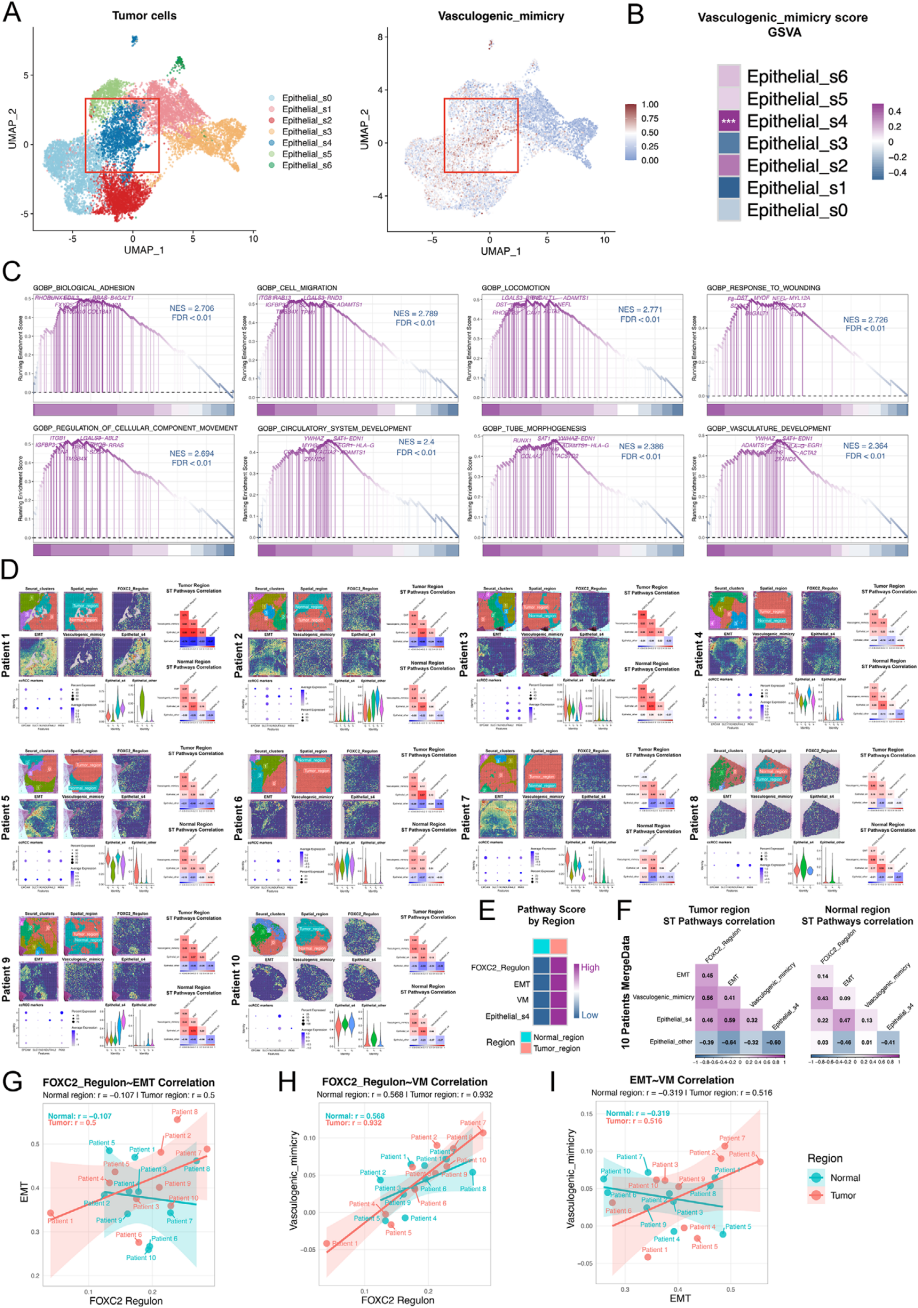
Spatial transcriptomics reveals the significant association of the Epithelial_s4 subpopulation with VM in renal cell carcinoma. (A),(B) Single‐cell data analysis demonstrates marked activation of VM pathways in the Epithelial_s4 subpopulation, significantly higher than in other subpopulations. (C) Gene Set Enrichment Analysis (GSEA) indicates significant enrichment of tube formation‐related pathways in the Epithelial_s4 subpopulation. (D) Spatial mapping and region‐specific analysis for 10 ccRCC patients. For each patient, the spatial analysis is comprised of four panels: the upper left section displays the dimensionality reduction and clustering results of spatial spots (Seurat_clusters), the identification of Tumor_region and Normal_region (Spatial_region), and the spatial enrichment patterns of FOXC2_Regulon, EMT, VM, and Epithelial_s4; the lower left panel includes a DotPlot showing the expression of key ccRCC marker genes (EPCAM, SLC17A3, NDUFA4L2, PAX8) across different spatial spot clusters, alongside violin plots depicting the distribution of Epithelial_s4 and Epithelial_other scores from RCTD deconvolution within these clusters; the right section visualizes the correlation matrices of pathway scores among spatial spots within the defined Tumor_region and Normal_region. (E) A heatmap demonstrating that the enrichment scores for FOXC2_Regulon, EMT, VM, and Epithelial_s4 are significantly higher in the Tumor_region compared to the Normal_region. (F) Correlation analysis of FOXC2_Regulon, EMT, VM, Epithelial_s4, and Epithelial_other was performed separately in the Tumor_region and Normal_region after merging spatial spots data from all 10 patients. (G)–(I) Patient‐level analysis of pathway co‐activation. The average activation scores for FOXC2_Regulon, EMT, and VM were calculated per patient within the Tumor_region and Normal_region, respectively. Their associations are displayed as Pearson correlation scatter plots. The correlation strength for each pair of pathways is markedly higher in the Tumor_region than in the Normal_region.

Previous studies have demonstrated that the synchronous activation of EMT and angiogenesis serves as a critical hallmark of tumor cells with robust VM capability [29, 30]. To further investigate VM activation across tumor cell subpopulations, our in‐depth analysis revealed that the Epithelial_s4 subpopulation exhibited prominent VM pathway activation signatures (Figure 2A,B). Notably, the Epithelial_s4 subpopulation exhibits significant upregulation of multiple genes closely associated with VM across various tumor subtypes, further implicating its critical role in the VM process (Figure S2K) [31, 32, 33, 34, 35, 36, 37, 38]. Concurrently, multiple VM‐associated pathways—including cell‐cell adhesion, cell migration, blood vessel morphogenesis, and tube formation—were significantly enriched in this subpopulation (NES > 2; FDR < 0.01) (Figure 2C).

We performed a region‐specific quantitative analysis of spatial transcriptomic data from 10 patients with ccRCC. Based on the expression profiles of ccRCC markers, we accurately delineated the Tumor and Normal regions in each patient (Figure 2D). Quantitative comparison revealed that the activities of EMT, VM, and Epithelial_S4 were significantly higher in the Tumor region than in the Normal region (Figure 2E).

Spatial correlation analysis within the tumor region further demonstrated a robust co‐activation trend among EMT, VM, and Epithelial_S4, with all four showing significant negative correlations with Epithelial_other. This coordinated expression pattern was consistently observed both in individual patients and in the merged dataset (Figure 2F). Patient‐level analysis indicated that the correlations among EMT, and VM were also significantly stronger in the Tumor region than in the Normal region (Figure 2I). Together, these results indicate that EMT, VM, and Epithelial_s4 are not only specifically enriched in the tumor region but also form a spatially co‐localization.

### 3.4 Gene Network Analysis Resolves the Features of Tumor Cell Subgroups

**FIGURE 3:**
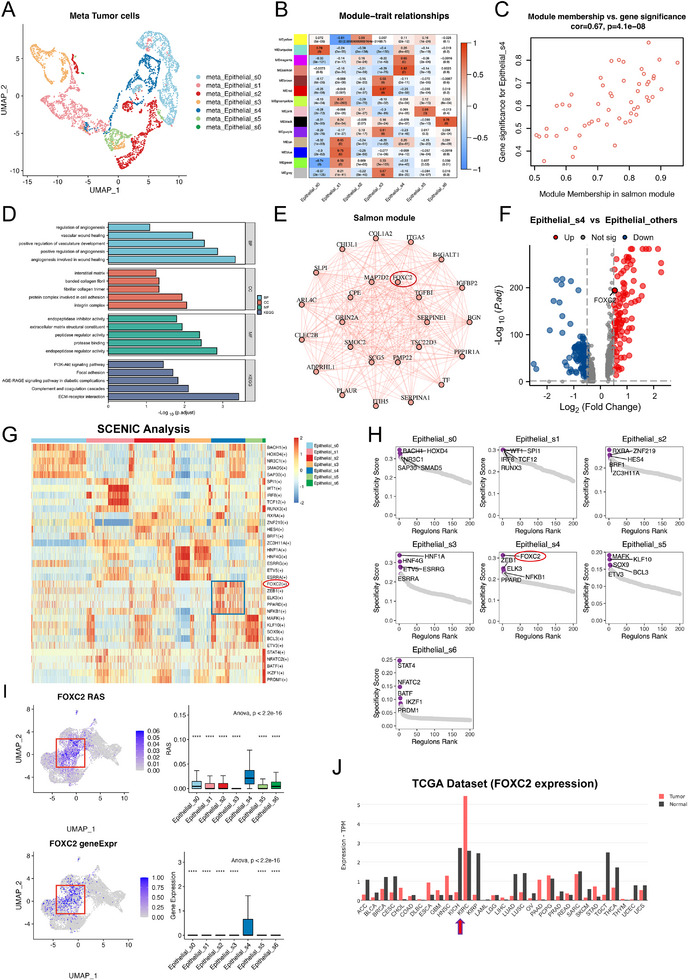
Multi‐dimensional analyses identify FOXC2 as the key transcription factor in the Epithelial_s4 subpopulation. (A) Integration of scRNA‐seq data into pseudobulk metacells via neighboring cell clustering to mitigate sparsity in the single‐cell matrix. Clustering results align with those from the original single‐cell data. (B),(C) scWGCNA analysis identifies 14 gene modules, with the salmon module showing significant correlation with the Epithelial_s4 subpopulation (cor = 0.67, *p* < 0.001). (D) GO and KEGG analyses reveal that the salmon module is enriched in biological processes such as tube formation and epithelial‐mesenchymal transition (EMT). (E) Co‐expression network of the salmon gene module. (F) Volcano plot of differentially expressed genes between Epithelial_s4 and other tumor subpopulations, highlighting FOXC2 as significantly upregulated in Epithelial_s4. (G) pySCENIC heatmap of highly activated transcription factors across tumor subpopulations, with FOXC2 being the most activated in Epithelial_s4. (H) pySCENIC analysis demonstrates specific activation of the transcription factor FOXC2 in the Epithelial_s4 subpopulation. (I) Transcription factor activity and gene expression levels of FOXC2 are significantly elevated in Epithelial_s4. (J) TCGA data confirm higher FOXC2 expression levels in renal carcinoma.

To further explore the relationship between tightly co‐expressed gene modules and phenotypic traits in cancer cell clusters, we performed scWGCNA. First, we used the scWGCNA algorithm to aggregate transcriptionally similar cells into pseudobulk metacells. We then reduced dimensionality and clustered the metacells via a Seurat object to check whether the heterogeneity of the different clusters of cells was preserved. After the aggregation process, the subtypes were still well separated in transcriptional space (Figure 3A).

Next, we performed routine WGCNA on different metacell clusters (Figure S3A). We identified 14 modules ranging in size from 53 to 466 genes, and 2989 genes were hierarchically clustered based on topological overlap (Figure 3B; Figure S3B,C). The salmon gene module was the most significantly associated with Epithelial_s4 cells (cor = 0.67, *p* < 0.0001) (Figure 3C; Figure S3D).

To further determine the biological function of Epithelial_s4 cells, we performed gene ontology (GO) and Kyoto Encyclopedia of Genes and Genomes (KEGG) enrichment analyses on 58 genes in the salmon gene module. Among the biological process (BP) terms, “angiogenesis involved in wound healing”, “positive regulation of angiogenesis”, “positive regulation of vasculature development”, etc., were significantly activated. The activation of terms such as “integrin complex” in the cellular component (CC) category and “endopeptidase regulator activity” in the molecular function (MF) category suggests that Epithelial_s4 cells promote extracellular matrix remodelling, which promotes cancer cell invasion, migration, and metastasis. In the KEGG analysis, we also observed the activation of “ECM‐receptor interaction” and “focal adhesion”, which also confirmed the above conclusion (Figure 3D). We also noticed the activation of the PI3K‐AKT signalling pathway, which is closely correlated with VM [39].

The above results all suggest that Epithelial_s4 cells have strong VM activity accompanied by angiogenic and EMT activation, which promotes the malignant progression of ccRCC.

### 3.5 FOXC2 Serves as the Key Transcription Factor in the Epithelial_s4

To identify the core regulatory networks of each cancer cell subpopulation, we employed pySCENIC to calculate the regulon activity score (RAS) across distinct cell clusters. Our analysis revealed that the transcription factors most significantly activated in the Epithelial_s4 subpopulation were FOXC2, ZEB1, ELK3, PPARD, and NFKB1 (Figure 3G,H). Among these, FOXC2 not only exhibited the strongest transcriptional activation signature in this subpopulation (Figure 3G,H) but also emerged as a hub gene within the scWGCNA co‐expression module specific to Epithelial_s4 (Figure 3E,I). Differential expression analysis further confirmed that FOXC2 serves as a hallmark transcription factor distinguishing Epithelial_s4 from other subpopulations (log2FC > 1, *p* < 0.05) (Figure 3F). ST data analysis further revealed distinct co‐localization and distribution correlations among FOXC2_regulon, EMT, VM, and Epithelial_s4 (Figure 2D–I).

To validate the clinical relevance of FOXC2 in ccRCC, we analyzed its pan‐cancer expression profile using the GEPIA2 database. The results demonstrated that FOXC2 was significantly upregulated in ccRCC (KIRC) tumor tissues compared with normal samples (Figure 3J). Based on this evidence, we conclude that FOXC2 acts as a critical regulator mediating the VM process in ccRCC.

### 3.6 FOXC2 is a Key Regulator of VM in Aggressive ccRCC

**FIGURE 4:**
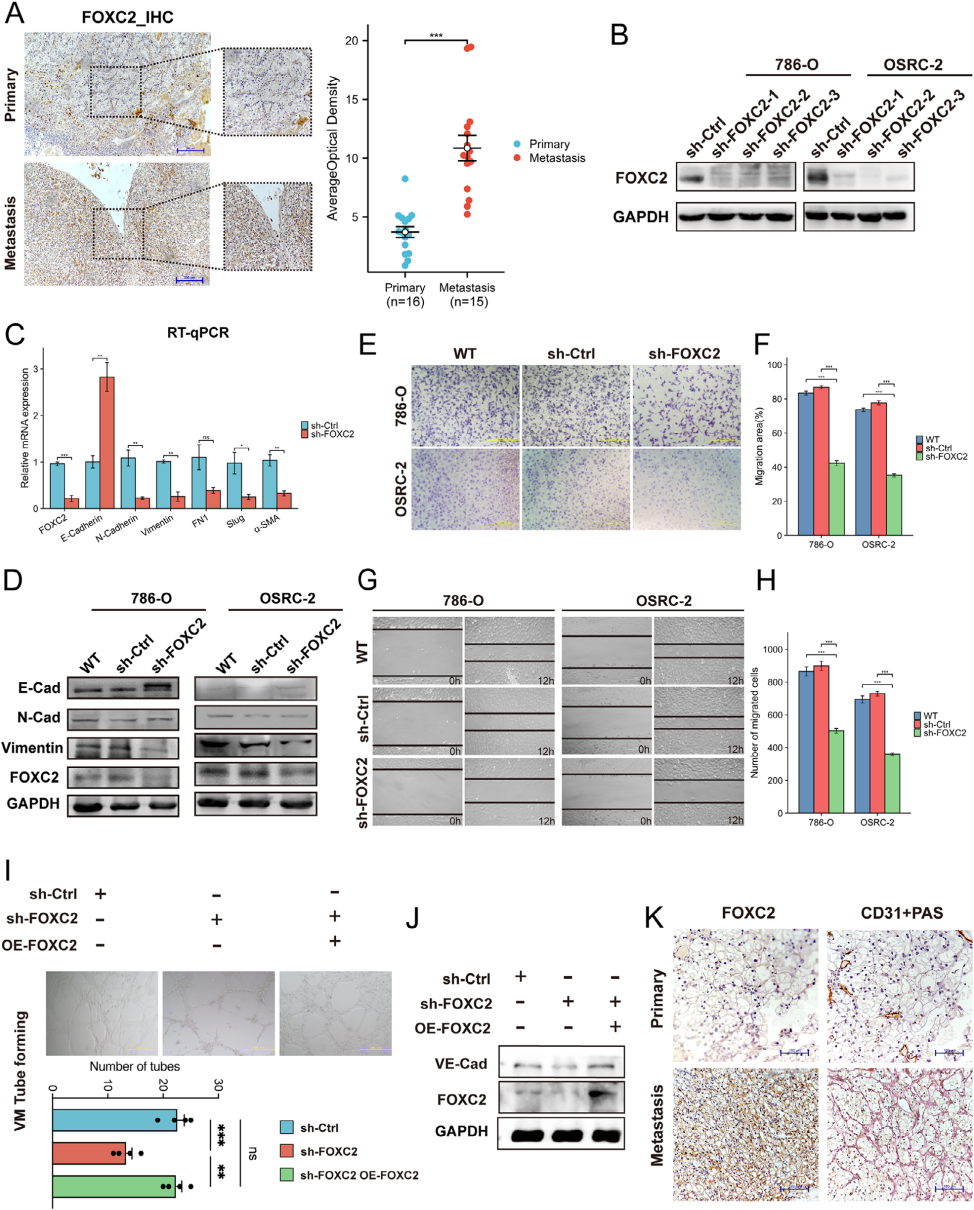
Experimental evidence elucidates the role of FOXC2 in regulating EMT and VM processes in ccRCC. (A) IHC analysis and quantitative comparison of FOXC2 protein expression in primary (n=16) vs. metastatic (n=15) ccRCC tissues (^**^ *p* < 0.01; original magnification ×200). (B) Establishment of FOXC2 knockdown models in 786‐O and OSCR‐2 cell lines, with Western blot validation of knockdown efficiency (sh‐Ctrl vs. sh‐FOXC2). (C) qPCR analysis of FOXC2 knockout effects on mRNA levels of EMT‐related genes (FOXC2, E‐cadherin, N‐cadherin, Vimentin, FN1, Slug, and α‐SMA) (n=3; ns *p* > 0.05, ^*^ *p* < 0.05, ^**^ *p* < 0.01, ^***^ *p* < 0.001). (D) Western blot analysis of core EMT markers (FOXC2, E‐cadherin, N‐cadherin, Vimentin) in wild‐type (WT), control (sh‐Ctrl), and FOXC2‐knockdown (sh‐FOXC2) groups. (E),(F) Transwell invasion assays showing suppressed invasive capacity of ccRCC cells upon sh‐FOXC2 group (n=3; ^***^ *p* < 0.001). (G),(H) Wound healing assays confirming significantly reduced migration ability in sh‐FOXC2 group (n=3; ^**^ *p* < 0.001). (I) Representative images of tube formation assays performed with 786‐O cells embedded in Matrigel. Cells were transfected with control shRNA (sh‐Ctrl), FOXC2‐targeting shRNA (sh‐FOXC2), or subjected to a rescue treatment with FOXC2 overexpression in the knockdown background (sh‐FOXC2 OE‐FOXC2). The number of tubes was quantified (ns: not significant, ^**^ *p* < 0.01, ^***^ *p* < 0.001). (J) Western blot analysis of FOXC2 and VE‐cadherin across three groups. (K) Serial section staining of metastatic vs. primary ccRCC tissues: FOXC2 IHC and CD31/PAS double staining illustrating vasculogenic mimicry distribution (original magnification ×200).

To validate the clinical relevance of the FOXC2, we assembled a cohort of ccRCC patients, comprising 16 primary tumors and 15 metastatic lesions resistant to post‐operative anti‐angiogenic therapy, and assessed FOXC2 expression by IHC. Quantitative analysis revealed that FOXC2 protein levels were significantly elevated in metastatic lesions compared to primary tumors (Figure 4A), reinforcing the association between FOXC2 and aggressive disease. Notably, FOXC2 was confirmed in the TCGA‐KIRC database to have clear correlations with EMT, angiogenesis, and VM‐related genes (Figure S4A–C). To further rule out potential interference from endothelial cells regarding FOXC2 expression, we performed additional characterization of endothelial subpopulations based on the scRNA‐seq data (Figure S4D,E). Analysis across tumor epithelial and endothelial subpopulations revealed that FOXC2 expression was highly enriched and specifically elevated in the Epithelial_s4 subpopulation (Figure S4F). These results clarify that FOXC2 is primarily localized to tumor cells and suggest that its function may be closely associated with specific tumor cell subpopulations that express this molecule (Figure S4G,H).

To further evaluate FOXC2's role in ccRCC metastasis, we first established stable knockdown cell lines (786‐O‐shFOXC2, OSRC‐2‐shFOXC2) via lentiviral infection (Figure 4B). Both RT‐qPCR and Western blot analyses confirmed that FOXC2 knockdown markedly reduced EMT phenotypes in ccRCC cells, evidenced by downregulation of EMT markers (N‐cadherin, Vimentin, and TGFβ) and upregulation of E‐cadherin (Figure 4C,D). Subsequent wound healing and Transwell migration assays demonstrated that FOXC2 silencing significantly impaired the migration and invasion capabilities of ccRCC cells (Figure 4E–G), with statistical significance determined by independent samples *t*‐tests (Figure 4F–H).

To investigate FOXC2's relationship with VM, we performed tumor cell tube formation assays. Results showed FOXC2 knockdown substantially inhibited VM formation in ccRCC cells, while FOXC2 reintroduction rescued this phenotype (Figure 4I; Figure S4I). Concurrently, FOXC2 knockdown led to a significant reduction in VE‐Cadherin (a VM marker) protein expression (Figure 4J). Furthermore, CD31/PAS double staining of clinical specimens revealed denser VM networks in tumor regions with high FOXC2 expression (Figure 4K).

These experimental results collectively demonstrate that FOXC2 plays a crucial regulatory role in promoting VM formation in ccRCC cells, thereby significantly contributing to advanced metastasis and therapy resistance.

### 3.7 Multi‐Omics Sequencing Identifies LAMA4 as a Key Downstream Effector of FOXC2

**FIGURE 5:**
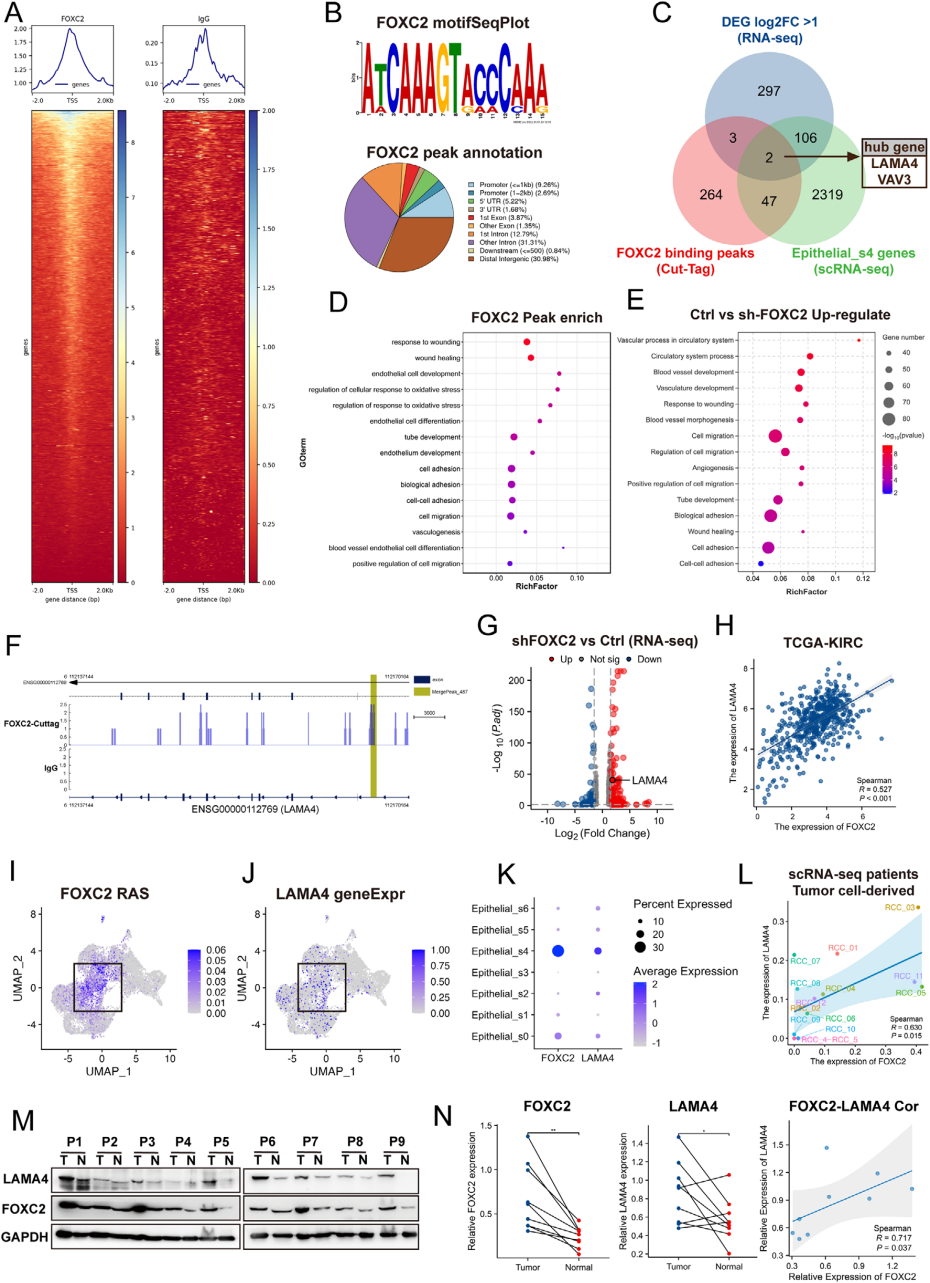
Multi‐omics sequencing identifies LAMA4 as a key downstream effector of FOXC2. (A) Genome‐wide binding signal heatmap of FOXC2 CUT&Tag sequencing in 786‐O cells (FOXC2 vs. IgG). (B) Distribution of FOXC2 binding peaks across the genome and motif analysis of its promoter‐bound sequences in 786‐O cells. (C) Venn diagram intersecting differentially expressed genes (log2FC > 1) upon FOXC2 knockdown, FOXC2‐bound downstream genes, and Epithelial_s4 subpopulation‐enriched genes, identifying LAMA4 and VAV3 as potential FOXC2 transcriptional targets. (D) GO/KEGG functional enrichment analysis of genes with FOXC2 binding peaks from CUT&Tag sequencing. (E) GO/KEGG functional enrichment analysis of RNA‐seq differentially expressed genes in FOXC2 knockdown vs. control groups. (F) CUT&Tag sequencing reveals a specific FOXC2 binding site (highlighted in dark yellow) within the upstream promoter region of the LAMA4 gene. (G) RNA‐seq data from 786‐O cells validate LAMA4 as a FOXC2‐regulated differentially expressed gene. (H) TCGA‐KIRC dataset analysis demonstrates a significant positive correlation between FOXC2 and LAMA4 mRNA expression (R = 0.527, ^**^ *p* < 0.01). (I)–(K) scRNA data show significant co‐expression of FOXC2 and LAMA4 in the Epithelial_s4 subpopulation. (L) scRNA data from 12 ccRCC patients confirm a robust expression correlation between tumor cell‐derived FOXC2 and LAMA4 (R = 0.630, ^*^ *p* < 0.05). (M) Western blot analysis of FOXC2 and LAMA4 protein levels in paired tumor and adjacent normal tissues from ccRCC patients. (N) Positive correlation between FOXC2 and LAMA4 protein expression. Protein levels were quantified by western blot (n=9; ^*^ *p* < 0.05, ^**^ *p* < 0.01). A significant correlation was found by Spearman analysis (R = 0.717, *p* = 0.037).

FOXC2's precise molecular regulatory mechanisms of VM remain incompletely understood [40]. To systematically analyze FOXC2's regulatory network, we first performed CUT&Tag sequencing (Figure 5A,B). To further identify FOXC2's functional targets, we integrated three datasets: RNA‐seq data from 786‐O cell models (Ctrl vs. sh‐FOXC2), expression profiles of the Epithelial_s4 subpopulation from single‐cell sequencing, and FOXC2's downstream regulatory gene set from CUT&Tag. Multi‐omics joint analysis identified LAMA4 and VAV3 as common target genes across all three datasets (Figure 5C). Both FOXC2‐target peaks and FOXC2‐KD DEG showed significant enrichment in biological processes related to VM, such as tube formation, cell adhesion, and vascular development (Figure 5D,E). Detailed analysis revealed that FOXC2 specifically binds to the promoter region of LAMA4, while primarily binding to the intronic region of VAV3. scRNA‐seq revealed that VAV3 was not specifically expressed in the Epithelial_s4 and showed no significant correlation associated with FOXC2 and VM function (Figure S6B,C). qPCR and dual‑luciferase reporter assays further demonstrated that neither overexpression nor knockdown of FOXC2 altered VAV3 expression or promoter activity (Figure S6D,E). Based on these findings, we selected LAMA4 as a target for downstream investigation.

To thoroughly investigate the FOXC2‐LAMA4 regulatory relationship, we conducted systematic validation experiments. CUT&Tag results showed significant peak enrichment of FOXC2 at the LAMA4 promoter region (Figure 5F), indicating direct regulation. RNA‐seq analysis of 786‐O cell models demonstrated significant LAMA4 downregulation following FOXC2 knockdown (log2FC >1, *P* <0.01) (Figure 5G). Analysis of TCGA‐KIRC datasets confirmed a strong positive correlation between FOXC2 and LAMA4 expression (R = 0.527, *p* < 0.01) (Figure 5H). scRNA‐seq revealed LAMA4's specific overexpression in Epithelial_s4 subpopulation cells (Figure 5I–K). Analysis of single‐cell data from 10 ccRCC patients showed a significant positive correlation between tumor cell‐derived FOXC2 and LAMA4 expression (R = 0.630, *p* < 0.01) (Figure 5L). Clinical sample validation by Western blot demonstrated significantly higher expression of both FOXC2 and LAMA4 in tumor tissues compared to adjacent normal tissues (Figure 5M). Importantly, the expression levels of FOXC2 and LAMA4 were found to be significantly positively correlated (R = 0.717, *p* = 0.037) (Figure 5N).

Collectively, these multi‐dimensional findings, combined with existing literature, establish LAMA4 as FOXC2's crucial intermediate effector in VM regulation, playing a pivotal role in ccRCC pathogenesis and progression.

### 3.8 Molecular Mechanism of FOXC2‐Mediated Transcriptional Regulation of LAMA4

**FIGURE 6:**
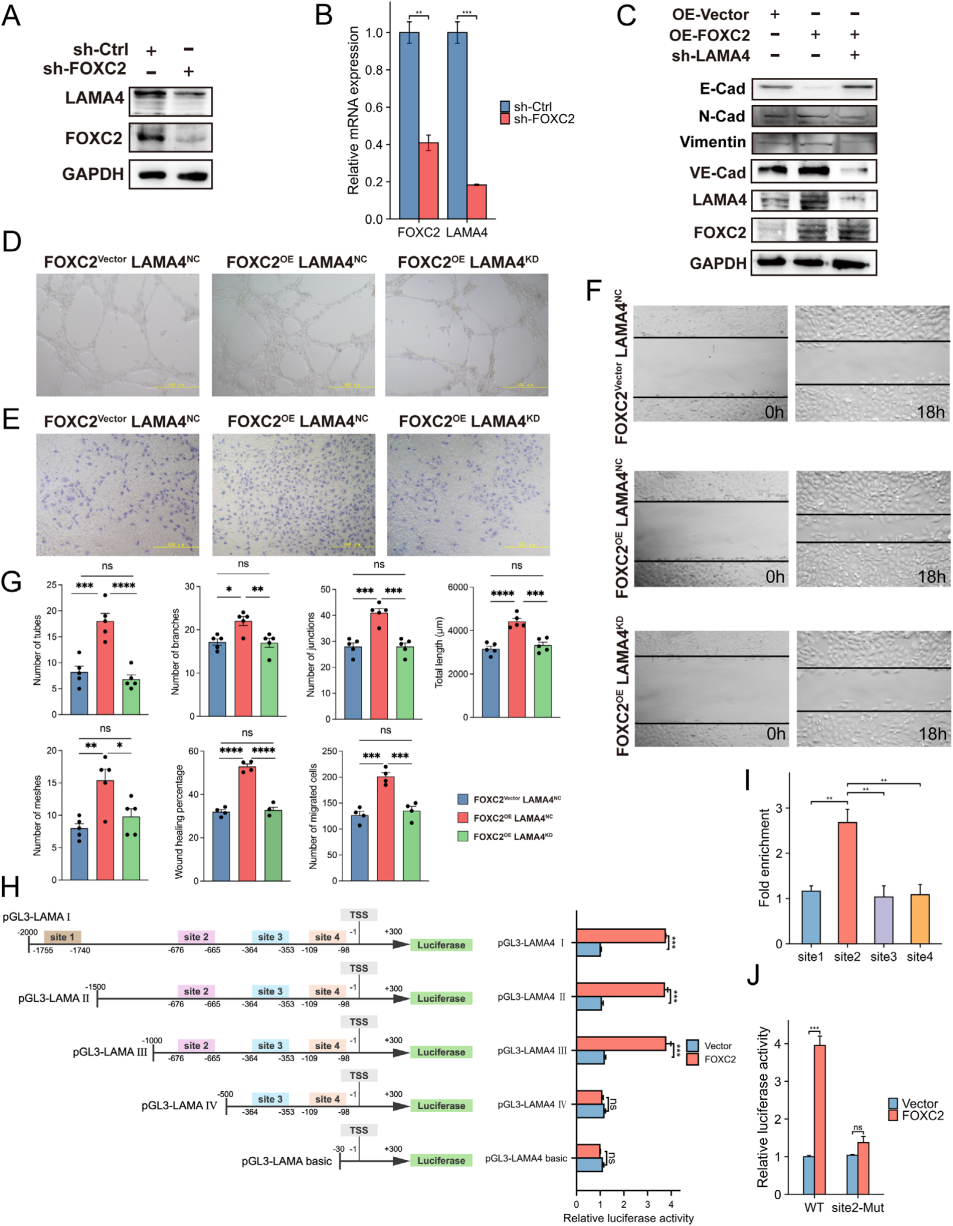
FOXC2 promotes VM in ccRCC cells by transcriptionally regulating LAMA4. (A) Western blot analysis of LAMA4 protein expression upon FOXC2 knockdown. (B) qPCR detection of LAMA4 mRNA levels following FOXC2 knockdown (n=3; ^**^ *p* < 0.01, ^***^ *p* < 0.001). (C) Western blot analysis of FOXC2, LAMA4, VE‑cadherin, and the EMT markers E‑cadherin, N‑cadherin, and Vimentin in FOXC2‑overexpressing (OE‐ FOXC2) and LAMA4‑knockdown (sh‐LAMA4) cell models. (D) Tube formation assays demonstrate that FOXC2 overexpression significantly enhances VM capacity in renal cancer cells, while LAMA4 knockdown partially reverses this effect (n=3; ^***^ *p* < 0.001). (E) Transwell invasion assays reveal that FOXC2 overexpression promotes cancer cell invasion, which is suppressed by LAMA4 knockdown (n=3; ^**^ *p* < 0.01, ^***^ *p* < 0.001). (F) Wound healing assays confirm that FOXC2 overexpression increases cell migration, an effect attenuated by LAMA4 knockdown (n=3; ^***^ *p* < 0.001). 0.001). (G) Statistical analysis was performed on the following metrics: tube formation (number of tubes, branches, junctions, meshes, and total length), wound healing (wound healing percentage), and Transwell (number of migrated cells). ns: not significant, ^*^ *p* < 0.05, ^**^ *p* < 0.01, ^***^ *p* < 0.001. (H) Luciferase reporter assays using LAMA4 promoter truncations transfected into renal cancer cells. FOXC2 regulates promoter activity across distinct LAMA4 promoter regions (n=3; ns *p* > 0.05, ^***^ *p* < 0.001). (I) ChIP with FOXC2 antibody followed by qPCR amplification of LAMA4 promoter fragments (site1–site4) (n=3; ns *p* > 0.05, ^**^ *p* < 0.01). (J) Luciferase assays comparing wild‐type and site2‐mutated LAMA4 promoter activity in renal cancer cells. FOXC2 selectively regulates wild‐type promoter activity (n=3; ns *p* > 0.05, ^***^ *p* < 0.001).

To elucidate the regulatory mechanism and functional relevance of FOXC2 on LAMA4, we conducted systematic molecular biology experiments. First, qPCR and Western blot analyses confirmed that FOXC2 knockdown significantly suppressed LAMA4 expression at both mRNA and protein levels (Figure 6A,B). To further investigate their functional relationship, we established stable 786‐O cell lines with FOXC2 overexpression and LAMA4 knockdown rescue. Western blot results indicated that FOXC2 overexpression significantly increased the expression of the VM marker VE‐cadherin and induced EMT‐related protein changes. Notably, these alterations were reversed upon LAMA4 knockdown, consistently supporting its role in mediating FOXC2's function (Figure 6C). Tube formation assays demonstrated that FOXC2‐promoted VM was dependent on LAMA4 expression (Figure 6D). Additionally, wound healing and Transwell migration assays showed that FOXC2‐mediated EMT in renal cancer cells also required LAMA4 participation (Figure 6E–G).

To dissect the molecular mechanism underlying FOXC2‐mediated regulation of LAMA4, we analyzed potential binding sites using the JASPAR database and identified four candidate sites in the LAMA4 promoter (site1: −1755 to −1740; site2: −676 to −665; site3: −364 to −353; site4: −109 to −98) (Figure S5A,B). We constructed a series of truncated LAMA4 promoter mutants (pGL3‐LAMA I; pGL3‐LAMA II; pGL3‐LAMA III; pGL3‐LAMA IV; pGL3‐LAMA basic). Dual‐luciferase reporter assays with these truncation plasmids revealed that FOXC2 primarily binds to site2 (−676 bp to −665 bp) of the LAMA4 promoter (Figure 6H). ChIP assays further confirmed significant FOXC2 binding enrichment at site2 (Figure 6I). Moreover, we generated pGL3‐LAMA4 site2‐Mut, containing a mutated FOXC2 binding sequence (−676 to −665). While co‐transfection of pGL3‐LAMA4 WT and FOXC2 overexpression plasmids significantly increased luciferase activity, this enhancement was rescued in cells co‐transfected with pGL3‐LAMA4 site2‐Mut and FOXC2 overexpression plasmids (Figure 6J).

These results collectively demonstrate that FOXC2 transcriptionally activates LAMA4 by directly binding to its promoter, thereby promoting VM formation and EMT progression in renal cancer cells.

### 3.9 LAMA4 Remodels the Metastatic Microenvironment by Promoting Metastasis‐Associated Macrophages (MAMs) Polarization in Pulmonary Metastases

**FIGURE 7:**
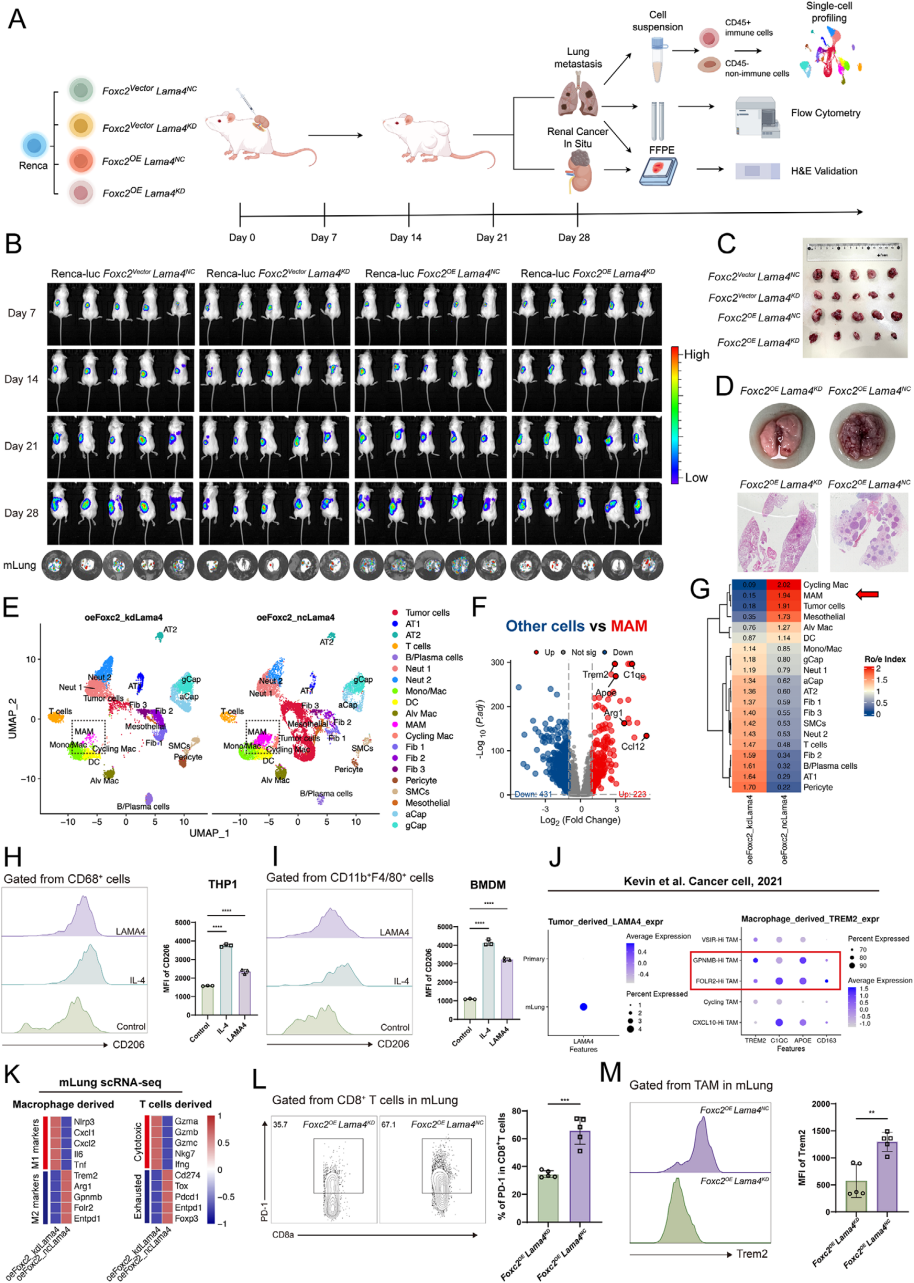
FOXC2‐LAMA4 remodels the metastatic microenvironment by promoting TREM2^+^ CD206^+^ MAM polarization in pulmonary metastases. (A) Experimental design and grouping for orthotopic renal carcinoma xenografts. (B) In vivo imaging system dynamically monitors renal orthotopic tumor growth (days 7, 14, 21, 28) and lung metastasis formation (terminal imaging at day 28) across groups. (C) Comparison of renal tumor volumes at the experimental endpoint among groups. (D) Lung metastasis imaging and H&E staining validation in the Foxc2 overexpression group (Foxc2^OE^ Lama4^NC^) vs. Foxc2 overexpression + LAMA4 knockdown group (Foxc2^OE^ Lama4^KD^). (E) Dimensionality reduction and cell type annotation of scRNA data from lung tissues of Foxc2^OE^ Lama4^NC^ and Foxc2^OE^ Lama4^KD^ mice. (F) Heatmap of MAMs signature genes showing upregulated immunosuppressive markers (e.g., Trem2, Arg1) (log2FC >1, ^*^ *p* < 0.05). (G)  Tissue preference analysis (R/oe score) highlights significant enrichment of MAMs (red arrow) in the Foxc2^OE^ Lama4^NC^ group. (H),(I) Flow cytometry analysis of the effect of LAMA4 on inducing immunosuppressive polarization. Shown is the MFI of CD206 (H) within the CD68^+^ population for human THP1‑derived macrophages and (I) within the CD11b^+^F4/80^+^ population for mouse BMDMs following treatment with LAMA4. Untreated cells and IL‑4‑treated cells served as negative and positive controls, respectively (n = 3, ^****^ *p* < 0.0001). (J) scRNA data from ccRCC lung metastases show high LAMA4 expression and elevated Trem2^+^ macrophage markers (TREM2, C1QC, APOE, CD163) in GPNMB‐Hi and FOLR2‐Hi macrophages. (K) scRNA of murine lung metastases: Macrophages in the oeFoxc2 ncLama4 group exhibit higher M2 markers, while T cells display pronounced exhaustion markers. (L),(M) Flow cytometry analysis of immune cell phenotypes in lung metastases from the orthotopic kidney cancer model. (L) Proportion of exhausted (CD8a^+^ PD‑1^+^) CD8^+^ T cells. (M) MFI of TREM2 on macrophages. Comparisons are between the FOXC2^OE^LAMA4^NC^and FOXC2^OE^LAMA4^KD^ groups (n = 5). (^**^ *p* < 0.01, ^***^ *p* < 0.001).

While previous studies have demonstrated that LAMA4 enhances tumor cell distant metastasis and colonization capacity [26], our research further uncovered the critical regulatory role of LAMA4 in the metastatic microenvironment. To investigate this, we established four orthotopic renal tumor models using RENCA‐Luc cells: control group (Foxc2^Vector^ Lama4^NC^), LAMA4 knockdown group (Foxc2^Vector^ Lama4^KD^), FOXC2 overexpression group (Foxc2^OE^ Lama4^NC^), and FOXC2 overexpression with LAMA4 knockdown group (Foxc2^OE^ Lama4^KD^) (Figure 7A; Figure S6A). We observed significant formation of pulmonary metastases in the Foxc2^OE^ Lama4^NC^ group by day 21, whereas the Foxc2^OE^ Lama4^KD^ group showed minimal metastatic foci (Figure 7B–D; Figure S7A). These findings suggest that LAMA4 significantly promotes renal cancer cell proliferation and pulmonary metastasis, while LAMA4 knockdown effectively inhibits this promoting effect.

To further elucidate the potential mechanism of the FOXC2‐LAMA4 axis in the renal cancer metastatic microenvironment, we performed scRNA‐seq analysis on lung metastatic tissues from Foxc2^OE^ Lama4^NC^ and Foxc2^OE^ Lama4^KD^ groups (Figure 7A). R/oe tissue preference analysis revealed that MAM infiltration was significantly higher in the Foxc2^OE^ Lama4^NC^ group compared to controls (Figure 7E,G). Detailed characterization of MAM showed they exhibited high Arg1 and Trem2 expression and displayed typical M2‐like immunosuppressive phenotypes (Figure 7F). We next investigated whether LAMA4 directly drives this immunosuppressive polarization. Treatment of both human THP‐1‐derived and BMDMs with recombinant LAMA4 protein significantly increased the surface expression of the M2 marker CD206. This effect was comparable to, though less pronounced than, that of the positive control IL‐4 (Figure 7H,I). At the transcriptional level, LAMA4 stimulation robustly upregulated classic immunosuppressive markers, including ARG1 and CD206 (Figure S7D–G), further confirming the induction of an immunosuppressive phenotype at the transcriptional level.

Integration of a published ccRCC lung metastasis single‐cell dataset (Cancer Cell, 2021) reinforced our conclusions [41]. Results showed that LAMA4 expression was significantly elevated in metastatic lung (mLung) tissues compared to primary lesions, and Trem2^+^ macrophage signature genes showed substantial overlap with known immunosuppressive TAM subsets (GPNMB‐hi and FOLR2‐hi) (Figure 7J). Further immune profiling of mouse lung metastases revealed that the Lama4 knockdown group exhibited upregulation of cytotoxic markers (Gzma, Gzmb, Ifng) and downregulation of exhaustion markers (Cd274, Tox, Pdcd1) in CD8^+^ T cells (Figure 7L). Concurrently, macrophages in this group showed increased M1 markers (Nlrp3, Tnf) and decreased M2 markers (Arg1, Trem2) (Figure 7K). Consistent with these findings, LAMA4 knockdown in vivo led to a significant reduction in both the proportion of exhausted CD8^+^ T cells (CD8a^+^PD‐1^+^) and the expression of the MAM marker TREM2 on macrophages within lung metastases (Figure 7L,M). Collectively, these data demonstrate that the FOXC2‐LAMA4 axis promotes metastasis by remodeling the immune microenvironment, primarily through the induction of TREM2^+^ CD206^+^ MAM, which in turn facilitates CD8^+^ T cell exhaustion and creates a permissive niche for metastatic outgrowth.

### 3.10 LAMA4‐ITGA6 Signaling Triggers p‐STAT6/GATA3‐Driven Macrophage Reprogramming

**FIGURE 8:**
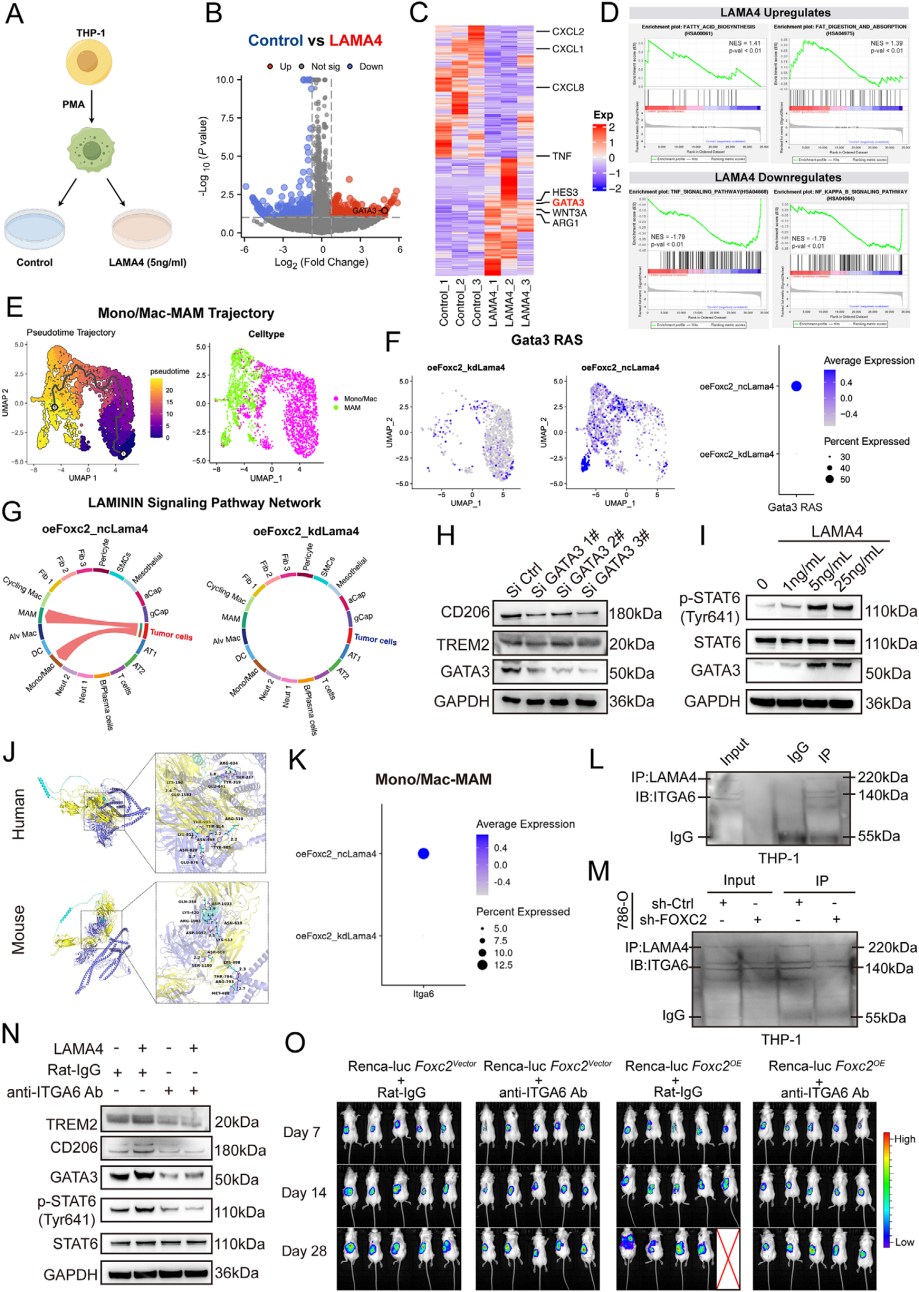
LAMA4‐ITGA6 binding activates STAT6 phosphorylation to drive GATA3‐dependent TREM2^+^ CD206^+^ MAM polarization, promoting metastatic outgrowth. (A) Schematic representation of the experimental setup to investigate the role of LAMA4 in macrophage polarization. THP‐1 cells were treated with PMA to differentiate into macrophages, followed by stimulation with LAMA4 (5 ng/mL). (B) Volcano plot comparing gene expression profiles between control and LAMA4‐treated THP‐1 macrophages. Red dots indicate upregulated genes; blue dots indicate downregulated genes. GATA3 was highly expressed in the LAMA4 treatment group. (C) Heatmap showing DEGs between control and LAMA4‐treated THP‐1 macrophages. Upregulated and downregulated genes are represented in red and blue, respectively. CXCL2, CXCL1, and TNF (inflammatory factors) exhibited elevated expression in controls, whereas ARG1, GATA3, and HES3 (immunosuppressive markers) showed significant upregulation in LAMA4‐treated macrophages. (D) GSEA demonstrating LAMA4‐mediated upregulation of the Fatty Acid Metabolic signaling pathway and downregulation of the TNF/NF‐κB signaling signaling pathway. (E) Pseudotime trajectory constructed by Monocle revealed that MAMs are derived from monocytes/macrophages (Mono/Mac) in mouse lung metastatic niches. (F) SCENIC analysis revealed heightened Gata3 regulon activity score (Gata3 RAS) enriched in MAMs, with concurrent elevation of Gata3 activity in Mono/Mac from oeFOXC2_ncLama4 mice group. (G) Cell‐cell communication analysis indicated that tumor cells in the oeFOXC2_ncLama4 group release enhanced LAMININ signals, which significantly activate downstream pathways in monocytes/macrophages (Mono/Mac), suggesting LAMA4‐mediated LAMININ signaling drives Mono/Mac differentiation into MAMs via specific receptor engagement. (H) Western blot analysis confirmed significant downregulation of GATA3 protein following siRNA‐mediated silencing, with concomitant reduction in TREM2 and CD206 expression. (I) Western blot analysis demonstrated that escalating LAMA4 concentrations (0, 1, 5, 25 ng/mL) induced progressive upregulation of immunosuppressive markers CD206 and TREM2 in macrophages. (J) Molecular docking of human and murine LAMA4 with ITGA6 reveals conserved binding capacity. Structures depict ITGA6 (cyan cartoon; extracellular domain in yellow) and LAMA4 (blue cartoon). (K) Comparative analysis of Itga6 expression in Mono/Mac and MAM populations within lung metastases revealed significantly elevated levels in oeFoxc2_ncLama4 vs. oeFoxc2_kdLama4 cohorts, suggesting enhanced responsiveness to LAMA4‐mediated signaling. (L) Validation of the interaction between LAMA4 and ITGA6. Lysates from co‐cultures of 786‐O renal carcinoma cells and THP‐1 macrophages were subjected to Co‐IP using an anti‐LAMA4 antibody, followed by immunoblotting with an anti‐ITGA6 antibody to confirm their direct binding.(M) FOXC2 knockdown attenuates the LAMA4‐ITGA6 interaction. Co‐IP was performed on lysates from co‐cultures of THP‐1 macrophages with 786‐O cells stably expressing either sh‐Ctrl or sh‐FOXC2, using an anti‐LAMA4 antibody. Western blot analysis for ITGA6 shows reduced complex formation upon FOXC2 knockdown. (N) STAT6‐neutralizing antibody (anti‐ ITGA6 Ab, 5 µg/mL) treatment significantly blocked LAMA4 (25 ng/mL)‐induced upregulation of p‐STAT6, GATA3, TREM2, and CD206 proteins in Western blot analysis. (O) In vivo imaging of orthotopic Renca‐luciferase Foxc2‐overexpressing (Renca‐luc Foxc2^oe^) renal tumors in BALB/c mice treated with ITGA6‐neutralizing antibody (10 mg/kg, i.v., weekly) vs. Rat‐IgG control, showing differential tumor progression and metastatic burden at days 7, 14, and 28 post‐implantations.

Previous results demonstrated that LAMA4 stimulation induces the conversion of M0 macrophages to the immunosuppressive MAM subtype. To dissect the underlying molecular mechanisms, we compared the gene expression profiles of LAMA4‐stimulated vs. unstimulated M0 macrophages via transcriptome sequencing (Figure 8A). Differential expression analysis revealed that LAMA4 stimulation shifted macrophages toward an immunosuppressive phenotype, characterized by significant downregulation of pro‐inflammatory cytokines (e.g., CXCL1, CXCL2, CXCL8, TNF) and upregulation of immunosuppression‐associated molecules (e.g., ARG1, GATA3, HES3, WNT3A) (Figure 8B,C). Further Gene Set Enrichment Analysis (GSEA) confirmed that LAMA4 treatment downregulated inflammation‐related pathways (e.g., TNF signaling pathway, NF‐κB signaling pathway) while activating lipid metabolism pathways associated with TREM2^+^ macrophages (often termed lipid‐associated macrophages [42]) in macrophages (Figure 8D).

We constructed a pseudotime differentiation trajectory for monocyte‐macrophages in mouse lung metastatic foci, revealing that MAMs predominantly originated from monocyte‐macrophage differentiation (Figure 8E). Notably, in Lama4 knockout mice, the number of MAMs was significantly reduced (Figure 8F), and the ability of tumor cells to remodel intercellular communication within the microenvironment via LAMININ signaling was markedly impaired (Figure 8G). Consistent with elevated GATA3 expression in macrophages after in vitro LAMA4 stimulation, Gata3 transcription factor activity was also significantly reduced upon Lama4 knockout (Figure 8F). Given that GATA3 is a reported key regulator of immunosuppressive macrophages and can be activated by phosphorylated STAT6 (p‐STAT6) [43, 44], we hypothesize that LAMA4 may activate GATA3 via p‐STAT6, thereby driving macrophage reprogramming.

To validate these findings, we generated a GATA3‐knockdown macrophage model of THP1. GATA3 knockdown significantly downregulated TREM2 and the M2 marker CD206 (Figure 8H). Stimulation with increasing LAMA4 concentrations (0, 1, 5, 25 ng/mL) induced dose‐dependent increases in GATA3 and p‐STAT6 protein expression (Figure 8I), further indicating GATA3's critical role in LAMA4‐induced polarization toward the TREM2^+^ immunosuppressive macrophage phenotype.

To elucidate the upstream pathways activating STAT6 signaling, we identified ITGA6 as a significantly interacting protein of LAMA4 based on co‐immunoprecipitation mass spectrometry results from prior studies [45]. Molecular docking confirmed strong binding capacity between human/murine LAMA4 and the ITGA6 extracellular domain (Figure 8J). Additionally, within the monocyte/macrophage‐MAM single cells data, Itga6 expression was higher in the oeFOXC2_ncLama4 group (Figure 8K). Co‐culture and Co‐IP experiments directly validated the interaction between LAMA4 and ITGA6 across cell types (Figure 8L; Figure S8A). Furthermore, knockdown of FOXC2 in tumor cells significantly diminished LAMA4 interaction with macrophage ITGA6 (Figure 8M). These data establish FOXC2 as a key upstream regulator that promotes the formation of the LAMA4‐ITGA6 complex specifically at the interface of tumor cell‐macrophage communication. To verify if LAMA4‐ITGA6 induces macrophage polarization via p‐STAT6, macrophages were treated with an ITGA6 neutralizing antibody (anti‐ITGA6 Ab, 5 µg/mL) [46]. anti‐ITGA6 Ab significantly suppressed TREM2 and CD206 expression while blocking p‐STAT6 induction (Figure 8M), confirming that LAMA4‐induced TREM2 expression and M2‐like polarization depend on ITGA6‐STAT6 signaling axis activation.

Collectively, these data establish a paracrine LAMA4‐ITGA6/p‐STAT6/GATA3 signaling axis, through which FOXC2^+^ tumor cells drive TREM2^+^ CD206^+^ MAM reprogramming to foster a metastatic niche.

### 3.11 Targeting LAMA4‐ITGA6 Suppresses Tumor Growth and Metastasis In Vivo

To evaluate the therapeutic relevance of the LAMA4–ITGA6 axis in vivo, we assessed the effect of ITGA6 blockade. Orthotopic tumor‑bearing mice with Renca‐luc FOXC2^OE^ were treated weekly with anti‑ITGA6 Ab. In vivo imaging revealed that anti‑ITGA6 treatment significantly reduced both primary tumor volume and lung metastasis compared with the control group (Figure 8B,N), confirming the role of LAMA4–ITGA6 signaling in establishing a FOXC2‑driven metastatic niche.

We further assessed the therapeutic potential of combining ITGA6 blockade with the standard‑of‑care agent Sunitinib. In an orthotopic model, the combination prolonged mean survival time to 40.4 days, compared to 37.2 days with sunitinib alone and 31.1 days with vehicle (Figure S8C,D). Although the group survival difference did not reach statistical significance, a consistent trend toward delayed mortality was observed. In a lung metastasis model that permitted extended observation (Figure S8E), the combination nearly abolished metastatic lesions (Figure S8H), as confirmed by in vivo imaging and histology (Figure S8F), and significantly extended survival vs. sunitinib monotherapy (Log‑rank p = 0.02). Together, these data suggest that co‑targeting ITGA6 and sunitinib may enhance therapeutic efficacy, particularly in controlling metastatic ccRCC.

## 4 Discussion

This study provides a comprehensive dissection of the TME in ccRCC through an integrated multi‐omics approach. We have systematically unraveled the cellular heterogeneity of the TME, identified a pivotal malignant epithelial subpopulation (Epithelial_s4) with high metastatic and VM potential, delineated the FOXC2‐LAMA4 transcriptional regulatory axis, and elucidated a novel mechanism of metastatic niche formation via macrophage reprogramming. Our findings offer profound insights into the molecular drivers of ccRCC progression and therapeutic resistance.

Within the heterogeneous ccRCC landscape, we discovered a coordinated activation of EMT and vascular development pathways specifically within the Epithelial_s4 subpopulation [47, 48]. This co‐activation pattern is critical, as EMT not only confers stem cell‐like properties and enhanced plasticity [49], but is also considered a key permissive factor for initiating VM [48]. Further investigation identified FOXC2 as the core transcription factor of this subpopulation. As a non‐classical EMT‐related gene, FOXC2 has a well‐established role in inducing and maintaining the mesenchymal state in tumor cells [50, 51]. The EMT process confers stem cell‐like properties upon tumor cells and enhances their multipotent differentiation potential, which is considered a key permissive factor for initiating VM [8, 9]. Furthermore, our study reveals that FOXC2 expression is not only associated with tumor cell invasiveness, but also strongly correlated with the ability to form tubular networks resembling vascular channels. This suggests that the FOXC2 in this cluster is specialized toward supporting morphogenic processes rather than solely promoting migratory or invasive behavior. A technical limitation of the absence of in vivo Matrigel plug assays, which has constrained our ability to precisely delineate the dynamics of VM. This represents a gap in the present work. Future studies employing more refined in vivo models will be necessary to validate and extend these findings.

Furthermore, our subsequent mechanistic investigation bridged the FOXC2 downstream regulatory gap [40]. Multi‐omics screening and validation determined that VAV3 was not a direct transcriptional target of FOXC2. Given its stronger phenotypic link to VM and direct regulatory evidence, LAMA4 was prioritized as the key downstream effector of FOXC2 in this study. FOXC2 directly binds to the LAMA4 promoter region (−676 to −665 bp) to transcriptionally regulate LAMA4, thereby driving VM formation. As a key member of the laminin family, LAMA4 is widely expressed in vascular basement membranes [52]. Studies have shown that LAMA4 deficiency leads to abnormal vascular basement membrane structure, manifested as reduced branching and irregular lumens [52, 53]. This suggests LAMA4 may similarly remodel extracellular matrix structures around tumor cells to provide physical support for VM formation. Moreover, as an activator of integrin receptors, LAMA4 can promote cell migration and directional alignment through FAK and PI3K/AKT signaling pathways, which are the crucial steps in tube formation [54, 55, 56]. However, the mechanisms by which LAMA4 promotes VM formation are likely more complex [57, 58], involving multiple coordinated pathways that warrant further investigation [59, 60].

The implications of the FOXC2‐LAMA4 axis extend beyond the primary tumor. VM networks are thought to provide direct conduits for systemic dissemination, promoting tumor cell dissemination and increasing the probability of distant metastasis [9, 61, 62]. However, successful metastatic outgrowth requires dynamic crosstalk between tumor cells and the microenvironment to evade immune surveillance and establish a growth‐favorable niche [5, 63]. In our orthotopic renal cancer model, LAMA4 significantly enhanced pulmonary metastasis formation, indicating that the FOXC2–LAMA4 axis functions beyond cell‑autonomous effects to actively remodel the metastatic microenvironment. While earlier studies have shown that LAMA4 promotes metastatic colonization, its role in microenvironmental regulation remained unclear. Here, we demonstrate that LAMA4 acts as a signaling molecule that reprograms macrophages in the metastatic niche. Through binding to its receptor ITGA6 on macrophages, LAMA4 triggers STAT6 phosphorylation and subsequent upregulation of GATA3, driving macrophage polarization toward a TREM2^+^ CD206^+^ MAMs. This population fosters an immune‑suppressive niche in the lungs, which facilitates metastatic colonization and outgrowth, as evidenced by impaired cytotoxic T‑cell function and a broadly immunosuppressive landscape under high LAMA4 conditions. Notably, LAMA4 overexpression was accompanied by a shift of T cells toward a Th2 transcriptional program within lung metastases, suggesting that the LAMA4–GATA3 axis may coordinately regulate both myeloid and lymphoid lineages to collectively shape a pro‑metastatic microenvironment. Intriguingly, this Th2 bias occurred without significant upregulation of cytokines such as IL‑4 and IL‑13, implying that LAMA4 modulates immune cell function through mechanisms that extend beyond the classical cytokine network (Figure S7B). Future studies are warranted to further dissect how this signaling network integrates diverse immune cells to systemically promote metastasis.

The confirmed LAMA4‐ITGA6 interaction provides a concrete molecular mechanism for how FOXC2‐expressing tumor cells can reprogram the tumor microenvironment. This paracrine signaling axis, from tumor‐derived LAMA4 to macrophage ITGA6, offers a plausible explanation for the observed induction of immunoinhibitory macrophage polarization and subsequent support of tumor progression. We also found the autocrine potential in cancer cells, future studies investigating therapeutic blockade of this interaction could further validate its translational relevance. Our animal treatment models suggest that combining Sunitinib with ITGA6 blockade may provide added therapeutic benefit in ccRCC, particularly in suppressing metastasis and extending survival. The observed effects, while varying between models, offer preliminary support for further investigation of this dual‐targeting strategy. We observed that the combination of Sunitinib with an anti‐ITGA6 antibody exhibited promising therapeutic efficacy in a low‐dose tail vein model, significantly inhibiting the seeding and reactivation of circulating tumor cells in lung metastases. This effect is particularly relevant for post‐surgical adjuvant therapy in ccRCC, as it targets the critical phase of metastatic initiation. Furthermore, the pronounced benefit of this combination suggests that anti‐ITGA6 treatment may exert effects beyond reprogramming macrophages toward an immunosuppressive phenotype. It likely influences other key steps of the metastatic cascade, including integrin‐mediated colonization and the subsequent proliferation and outgrowth of disseminated tumor cells. Future studies exploring therapeutic agents are warranted to translate these findings into clinical benefit.

In summary, this study systematically elucidates the functional and mechanistic chain linking intratumoral cellular heterogeneity to distant organ metastasis in ccRCC. We propose a FOXC2–LAMA4‐driven metastatic cascade. Importantly, therapeutic validation targeting this axis demonstrates that antibodies against the LAMA4 receptor ITGA6 effectively inhibit primary tumor growth and lung metastasis in preclinical models and exhibit potential for enhanced anti‑metastatic efficacy when combined with sunitinib, thereby providing a direct therapeutic rationale for targeting this pro‑metastatic pathway.

## Funding

This work was supported by the National Key R&D Program of China (Grant No. 2023YFC2507000), the National Natural Science Foundation of China (Grant No. 82573157), the Shenyang Science and Technology Plan Project (Grant No.24‐214‐3‐21), the Science and Technology Planning Project of Liaoning Province of China (Grant No. 2023JH2/20200090).

## Conflicts of Interest

The authors declare no conflicts of interest.

## Supporting information




**Supporting File 1**: advs74155‐sup‐0001‐FigureS1‐S8.docx.

**Supporting File 2**: advs74155‐sup‐0002‐TableS1.xlsx.

**Supporting File 3**: advs74155‐sup‐0003‐TableS2.xlsx.

**Supporting File 4**: advs74155‐sup‐0004‐TableS3.xlsx.

## Data Availability

The datasets generated and analyzed during the current study are not publicly available but are available from the corresponding author upon reasonable request.
