# Supplementary material for: The FOXC2‐LAMA4 Axis Orchestrates Vasculogenic Mimicry and Immunosuppressive Niche Formation to Drive Metastatic Cascade in Renal Cell Carcinoma
Source: Adv Sci (Weinh). 2026 Jan 29;13(20):e16382. doi: 10.1002/advs.202516382 (PMC13067797; doi:10.1002/advs.202516382)
Supplement: Supplementary file 1 — Supporting File 1: advs74155‐sup‐0001‐FigureS1‐S8.docx. [file ADVS-13-e16382-s002.docx]

**
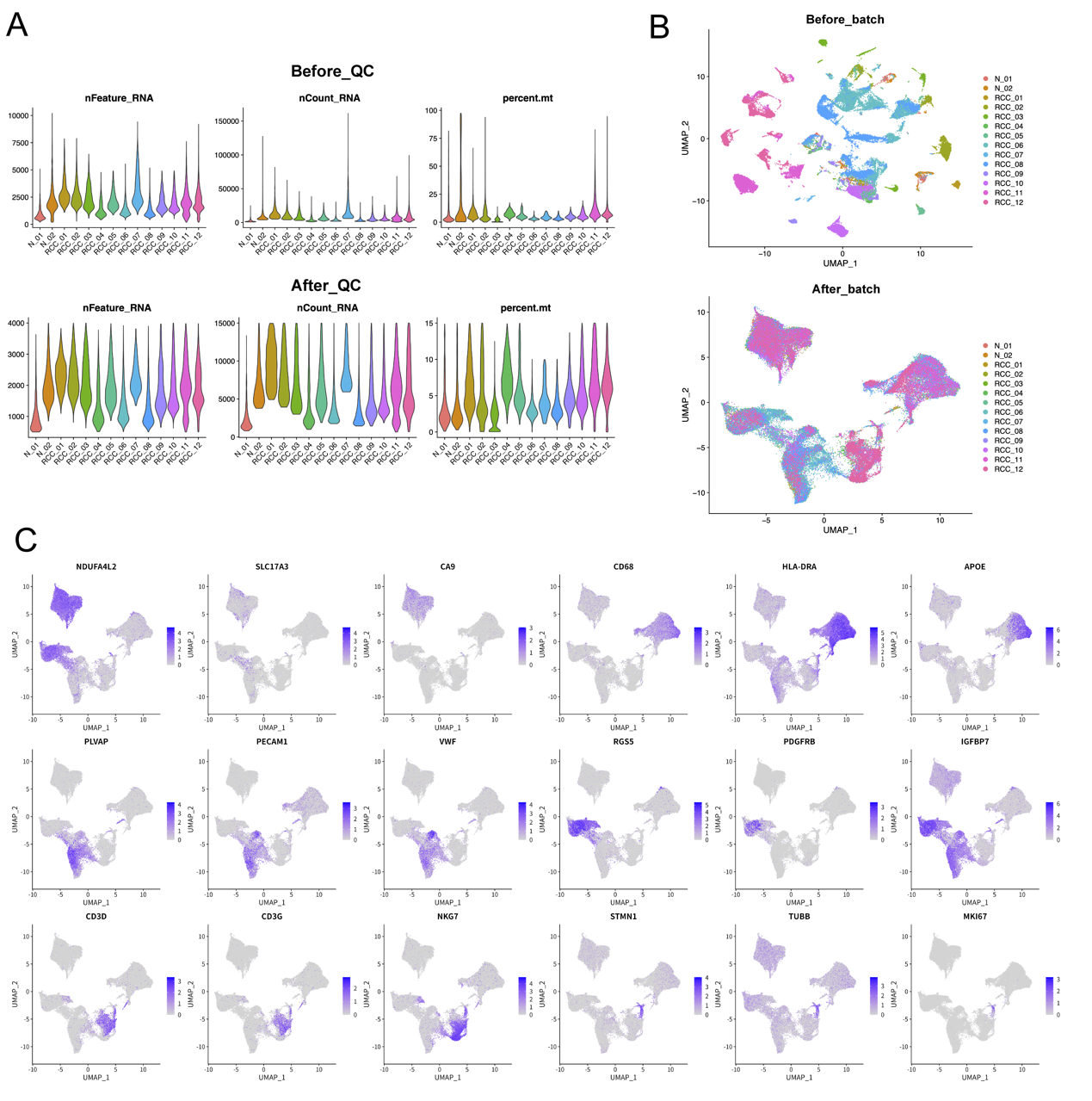
**

**Figure S1.** (A) Quality control (QC) of genes (nFeature_RNA), number of unique molecular identifiers (nCount_RNA) and percent of mitochondrial genes (percent.mt) per sample. (B) Comparison of the cell distribution before and after correcting for batch effects. (C) FeaturePlot illustrating marker gene expression across cell subpopulations.

**
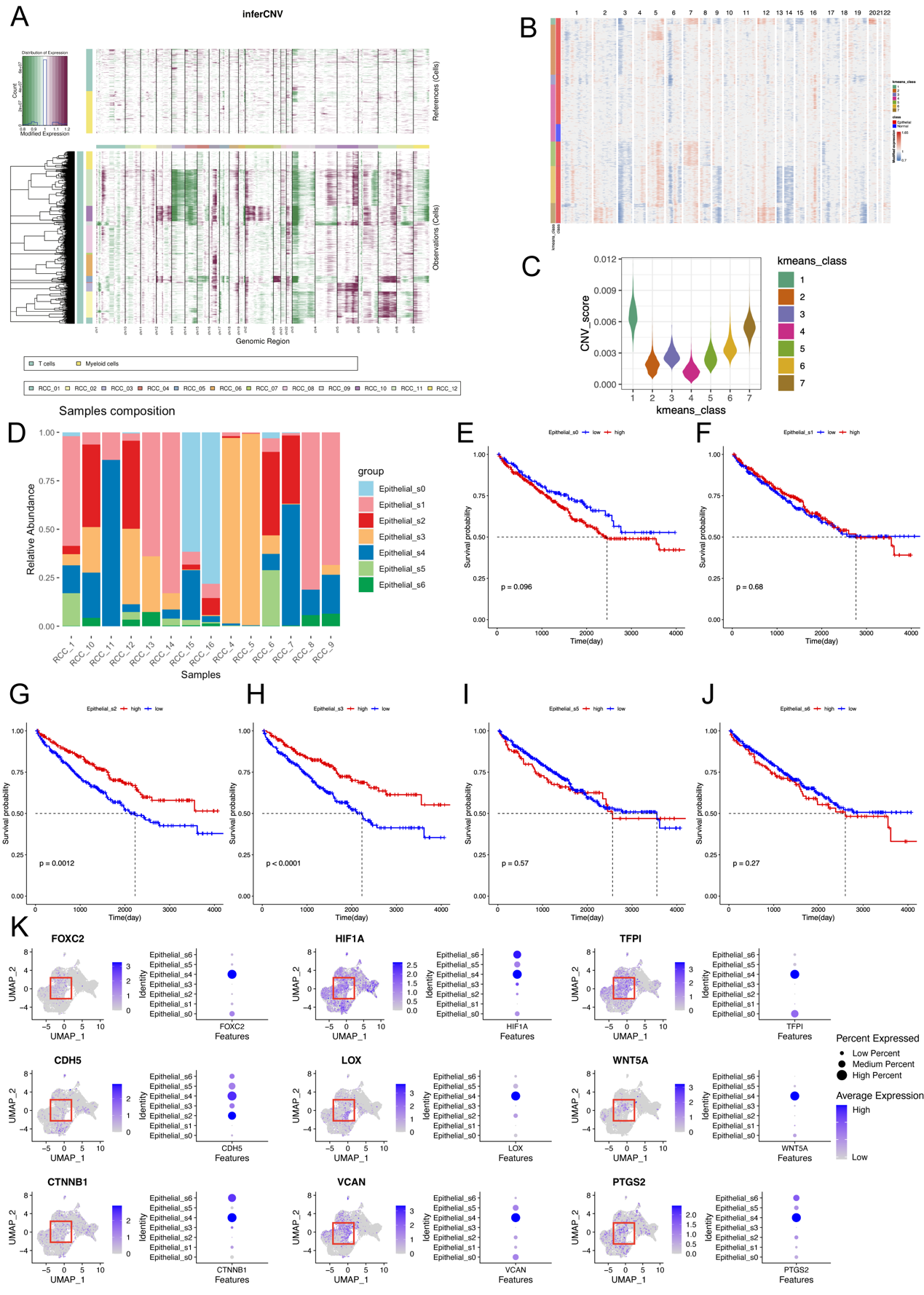
****Figure S2.** (A) InferCNV heatmaps of all epithelial cells grouped by different samples. (B) K-means clustering of epithelial cell CNVs with normal cells as a reference. (C) CNV scores per kmeans_class. (D) Proportions of tumor cell subpopulations across all patients. (E-J) Kaplan-Meier survival curves comparing overall survival between patients with high vs. low proportions of Epithelial_s0-s6 tumor cell subpopulations. (K) VM-associated genes expression (FOXC2, HIF1A, TFPI, CDH5, LOX, WNT5A, CTNNB1, VCAN, PTGS2) was evaluated across all tumor cell clusters. Red box highlights the Epithelial_S4 subpopulation in the UMAP.**
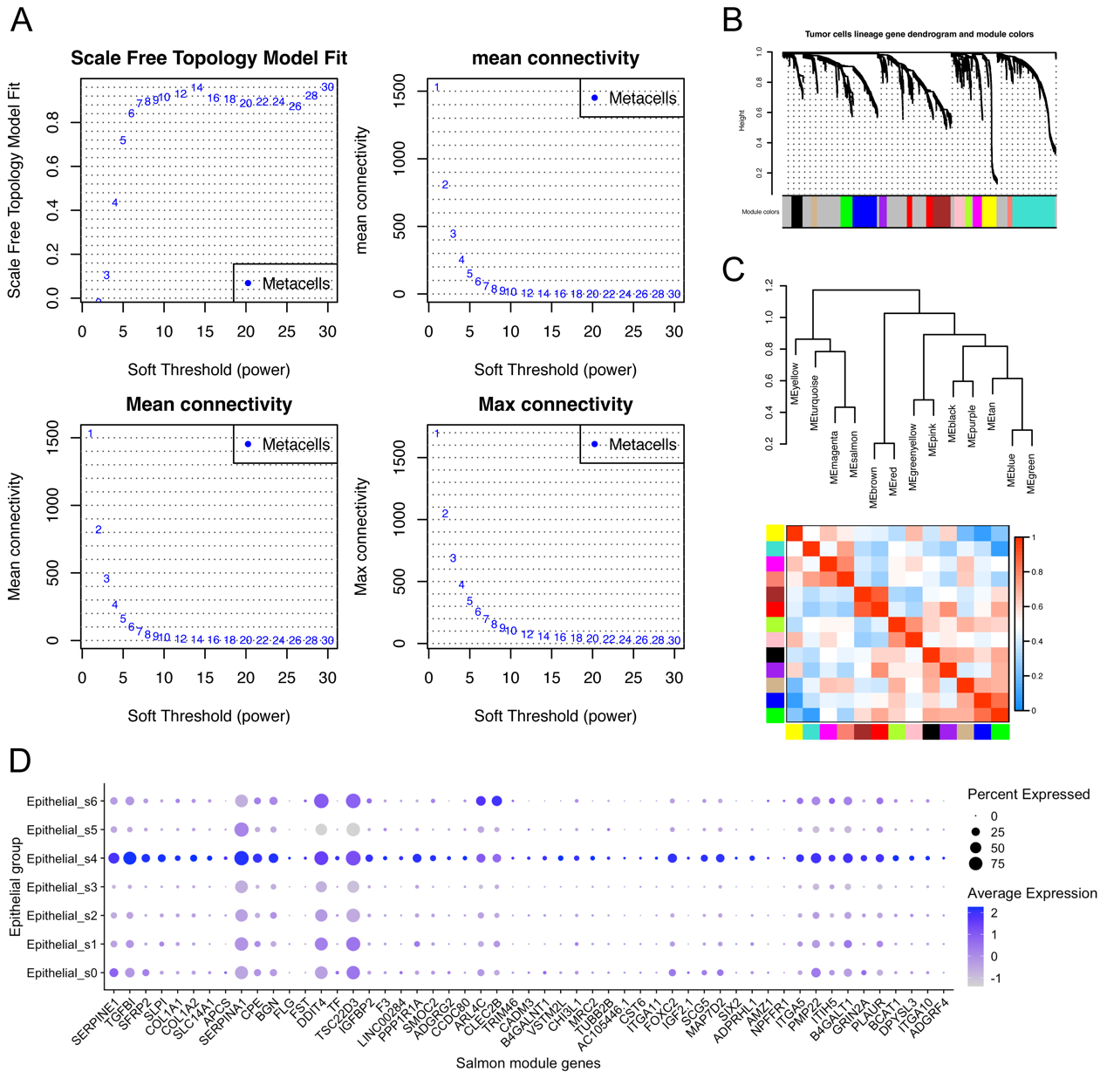
**

**Figure S3.** **(A)** Determination of the soft thresholding power via scale-free topology fitting index analysis. **(B)** WGCNA gene dendrogram. **(C)** Heatmap of adjacency relationships in the trait-weighted eigengene network, with red indicating higher inter-module correlations and blue indicating lower correlations. **(D)** Expression levels of Salmon Module genes across tumor cell subpopulations, with Epithelial_s4 showing significant enrichment of these genes.

**
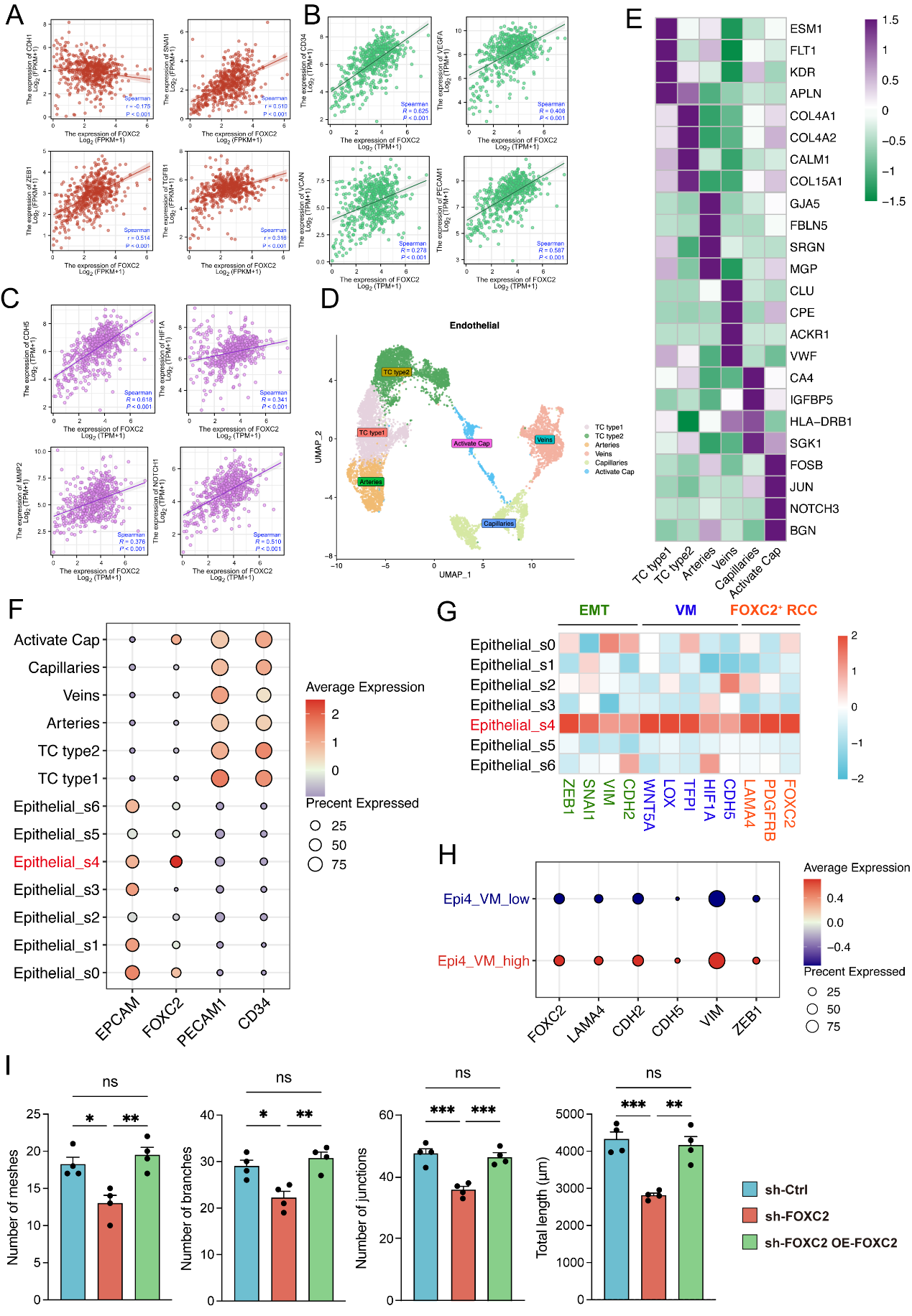
**

**Figure S4. (A)** Correlation analysis of FOXC2 RNA expression with EMT-related genes in the TCGA-KIRC dataset. **(B)** Correlation analysis of FOXC2 RNA expression with angiogenesis-related genes in the TCGA-KIRC dataset. **(C)** Correlation analysis of FOXC2 RNA expression with VM-related genes in the TCGA-KIRC dataset. **(D)** Further characterization of endothelial cell subpopulations from scRNA-seq data. Dimensionality UMAP reduction, clustering, and annotation of endothelial cell subsets. **(E)** Expression of selected marker genes defining each endothelial subpopulation. **(F)** Validation of key markers across tumor epithelial (Epithelial_s1–s6) and endothelial subpopulations: the epithelial marker EPCAM, FOXC2, and the endothelial markers PECAM1 and CD34. Note the specific high expression of FOXC2 in the Epithelial_s4 subpopulation. **(G)** Expression pattern of EMT-related genes (CDH2, VIM, SNAI1, ZEB1) and VM-related genes (CDH5, HIF1A, TFPI, LOX, WNT5A) across tumor cell subpopulations in scRNA-seq data. The Epithelial_s4 subpopulation exhibited distinct concurrent activation of both EMT and VM, along with high expression of marker genes such as FOXC2 and LAMA4.

**(H)** The Epithelial_s4 subpopulation was divided into high (Epi4_VM_high) and low (Epi4_VM_low) groups based on the median VM score. The Epi4_VM_high group showed significantly elevated expression of the FOXC2-LAMA4 signature, as well as VM- and EMT-related genes, supporting the identification of Epithelial_s4 as a tumor cell subpopulation with co-activated EMT and VM programs. **(I)** Representative images of tube formation assays performed with 786-O cells embedded in Matrigel. Cells were transfected with control shRNA (sh-Ctrl), FOXC2-targeting shRNA (sh-FOXC2), or subjected to a rescue treatment with FOXC2 overexpression in the knockdown background (sh-FOXC2 OE-FOXC2). The number of meshes, branches, junctions, and the total tube length were quantified (ns: not significant, *p < 0.05, **p < 0.01, ***p < 0.001).


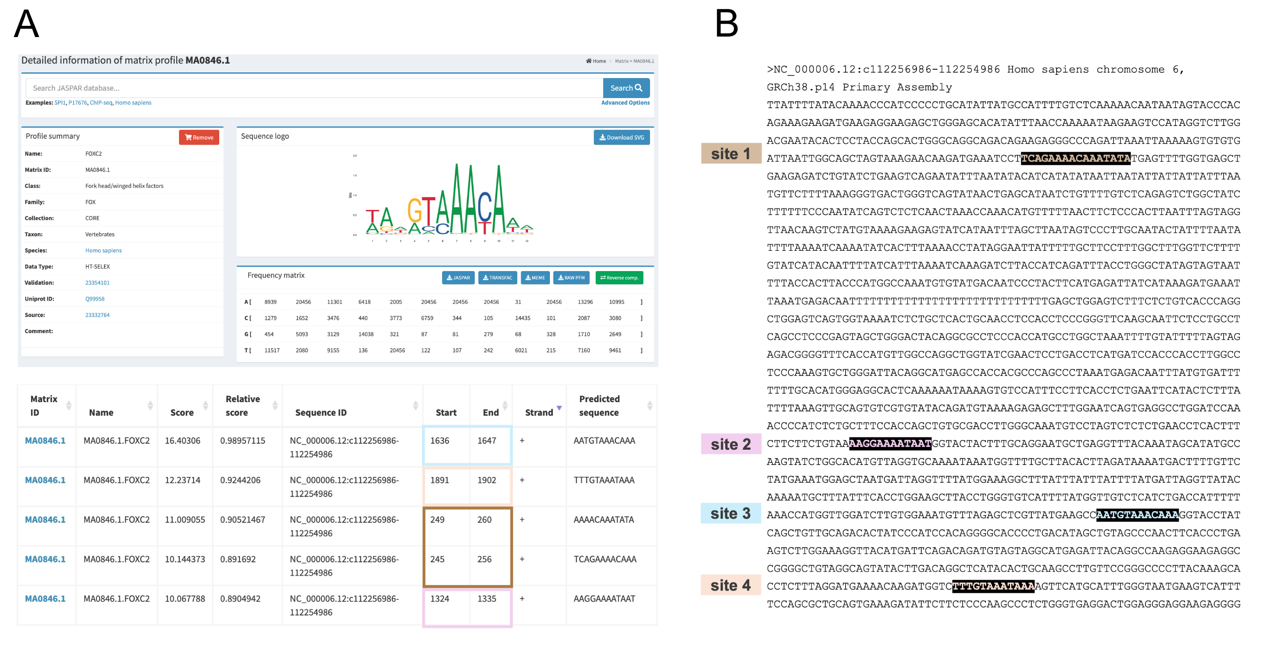


**Figure S5.** (A) JASPAR database prediction of potential FOXC2 binding motifs (Score > 10) within the LAMA4 promoter region. (B) Schematic representation of FOXC2 binding sites (site1-site4) in the LAMA4 promoter.


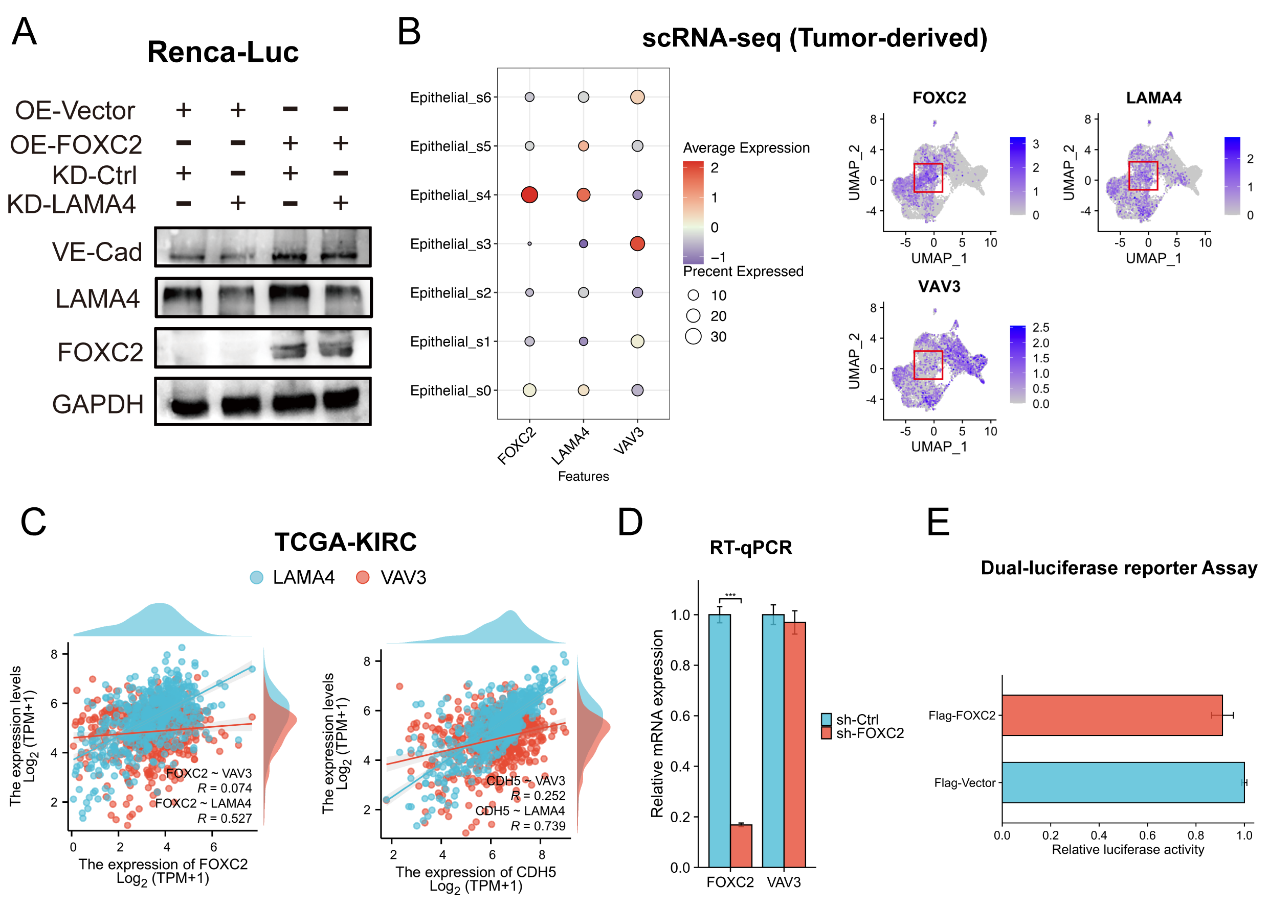


**Figure S6.** **(A)** Western blot analysis of FOXC2, LAMA4, and VE-cadherin protein levels in Renca cell lines across experimental groups. **(B)** Expression levels of FOXC2, LAMA4, and VAV3 across tumor epithelial subpopulations in single-cell data from ccRCC patients. FOXC2-high Epithelial_s4 subpopulation did not exhibit significant VAV3 expression. **(C)** Correlation analysis between LAMA4 and VAV3 with FOXC2 and the vasculogenic mimicry marker VE-Cadherin (CDH5) in the TCGA-KIRC database. **(D)** VAV3 expression levels before and after FOXC2 knockdown in the 786-O cell line, showing no significant trend of change. **(E)** A dual-luciferase reporter assay assessing the effect of FOXC2 on VAV3 promoter activity: Co-transfection of a reporter vector containing the VAV3 upstream promoter region (-2000 to 0) with a FOXC2 overexpression plasmid showed that FOXC2 overexpression did not significantly alter luciferase activity.


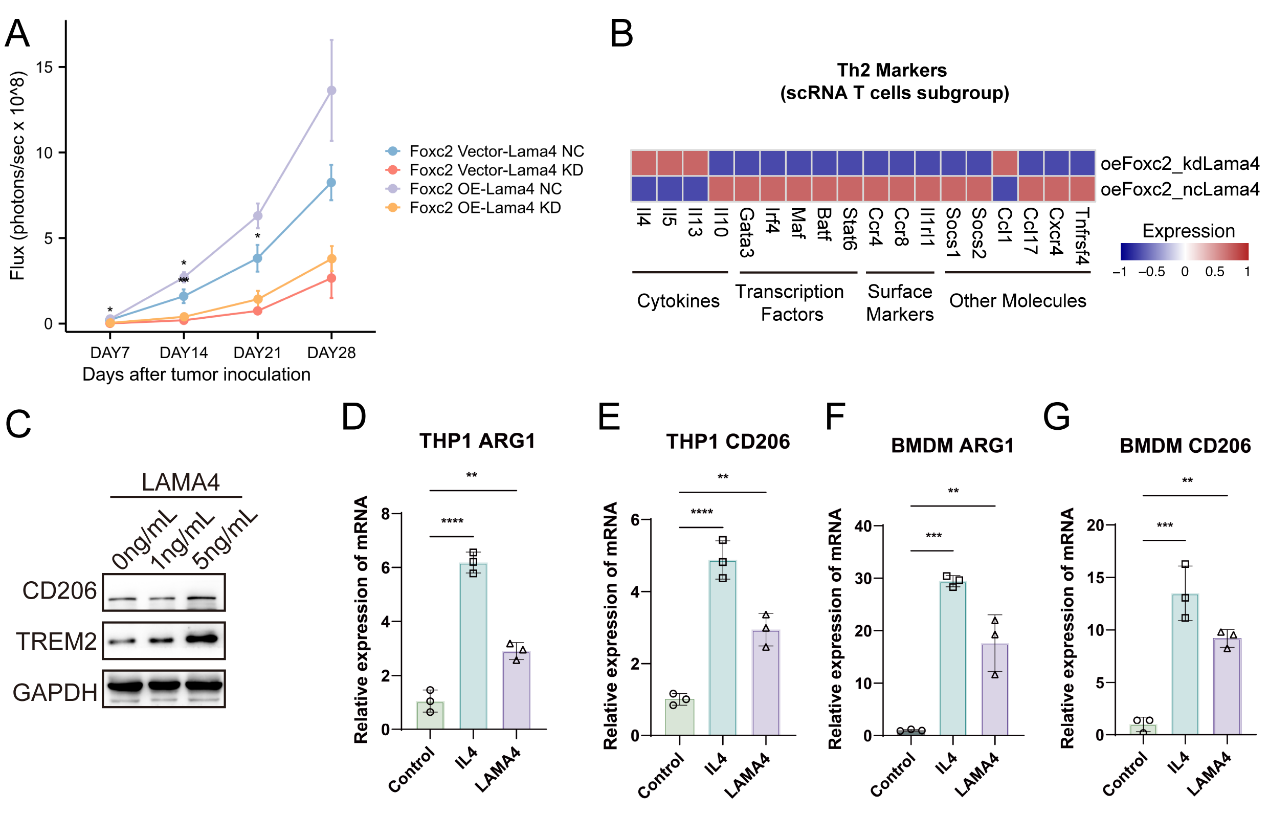


**Figure S7.** **(A)** In vivo imaging system monitoring orthotopic kidney tumor growth in mice at days 7, 14, 21, and 28 (n=5; **p < 0.05,* ***p < 0.01, ***p < 0.001*). **(B)** Evaluation of Th2-associated molecules in T cell subsets from scRNA-seq data of a mouse lung metastasis model. The assessed molecules include cytokines (Il4, Il5, Il13, Il10), transcription factors (Gata3, Irf4, Maf, Batf, Stat6), surface markers (Ccr4, Ccr8, Il1rl1), and other related molecules (Socs1, Socs2, Ccl1, Ccl17, Cxcr4, Tnfrsf4). **(C)** Western blot confirms upregulation of CD206 and TREM2 protein expression in THP-1 cells under LAMA4 gradient stimulation. **(D, E)** RT-qPCR analysis of ARG1 (D) and CD206 (E) expression in THP1 cells under indicated conditions: untreated control, IL‑4 (positive control), and LAMA4 treatment. **(F, G)** qPCR analysis of ARG1 (F) and CD206 (G) expression in BMDMs under the same treatment conditions. (n = 3; **p < 0.01, ***p < 0.001).


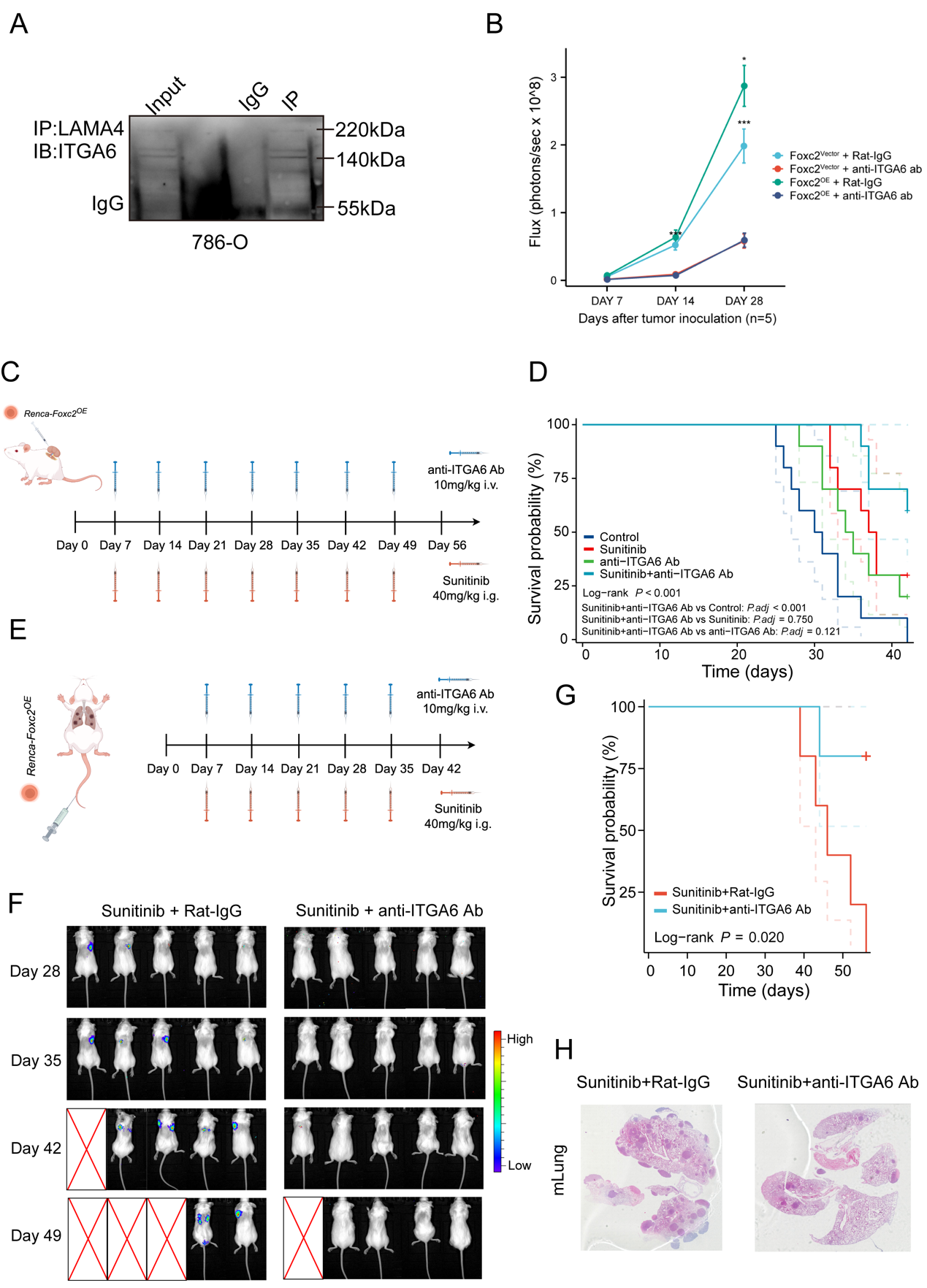


**Figure S8.** **(A)** Lysates from 786-O cells co-cultured with themselves were used for Co-IP with an anti-LAMA4 antibody, followed by immunoblotting with an anti-ITGA6 antibody to confirm direct binding. **(B)** In vivo imaging system monitoring orthotopic kidney tumor growth in mice at days 7, 14, and 28 (n=5; **p < 0.05, ***p < 0.001*) **(C)** Schematic diagram of the treatment regimen in an orthotopic renal carcinoma model. BALB/c mice bearing Renca‑Foxc2^OE^ tumors were treated with Sunitinib (oral gavage, 40 mg/kg, 5 days on/2 days off) and/or anti‑ITGA6 antibody (intravenous, 10 mg/kg, weekly). **(D)** Kaplan‑Meier survival curves of mice in the orthotopic model across four treatment groups: vehicle control, sunitinib monotherapy, anti‑ITGA6 monotherapy, and combination therapy. Survival was compared using the log‑rank test (P < 0.001), group survival statistical significance are displayed in the lower left panel. **(E)** Schematic of the low‑dose experimental lung metastasis model established via tail‑vein injection of Renca‑Foxc2^OE^ cells. This model allowed extended observation of metastatic progression and treatment response. **(F)** Survival analysis of the tail‑vein metastasis model comparing sunitinib monotherapy versus sunitinib combined with anti‑ITGA6 Ab. The combination significantly prolonged survival (log‑rank P = 0.02). **(G)** In vivo imaging of lung metastases over time. Images illustrate markedly reduced metastatic signal in the combination treatment group compared to Sunitinib alone. **(H)** Representative H&E‑stained lung sections from the tail‑vein model. The combination treatment group showed nearly complete suppression of metastatic nodules.
